# Venetoclax and pegcrisantaspase for complex karyotype acute myeloid leukemia

**DOI:** 10.1038/s41375-020-01080-6

**Published:** 2020-11-16

**Authors:** Ashkan Emadi, Bandish Kapadia, Dominique Bollino, Binny Bhandary, Maria R. Baer, Sandrine Niyongere, Erin T. Strovel, Hannah Kaizer, Elizabeth Chang, Eun Yong Choi, Xinrong Ma, Kayla M. Tighe, Brandon Carter-Cooper, Blake S. Moses, Curt I. Civin, Anup Mahurkar, Amol C. Shetty, Ronald B. Gartenhaus, Farin Kamangar, Rena G. Lapidus

**Affiliations:** 1 University of Maryland Marlene and Stewart Greenebaum Comprehensive Cancer Center, Baltimore, MD, USA; 2 Department of Medicine, University of Maryland School of Medicine, Baltimore, MD, USA; 3 Department of Pharmacology, University of Maryland School of Medicine, Baltimore, MD, USA; 4 Department of Pediatrics, University of Maryland School of Medicine, Baltimore, MD, USA; 5 University of Maryland Center for Stem Cell Biology & Regenerative Medicine, Baltimore, MD, USA; 6 Department of Physiology, University of Maryland School of Medicine, Baltimore, MD, USA; 7 Institute of Genome Sciences, University of Maryland, Baltimore, MD, USA; 8 Department of Biology, School of Computer, Mathematical, and Natural Sciences, Morgan State University, Baltimore, MD, USA; 9 Present address: Department of Internal Medicine, Virginia Commonwealth University School of Medicine, Hunter Holmes McGuire Veterans Affairs Medical Center, Richmond, USA

## Abstract

Complex karyotype acute myeloid leukemia (CK-AML) has a dismal outcome with current treatments, underscoring the need for new therapies. Here, we report synergistic anti-leukemic activity of the BCL-2 inhibitor venetoclax (Ven) and the asparaginase formulation Pegylated Crisantaspase (PegC) in CK-AML in vitro and in vivo. Ven-PegC combination inhibited growth of multiple AML cell lines and patient-derived primary CK-AML cells in vitro. In vivo, Ven-PegC showed potent reduction of leukemia burden and improved survival, compared with each agent alone, in a primary patient-derived CK-AML xenograft. Superiority of Ven-PegC, compared to single drugs, and, importantly, the clinically utilized Ven-azacitidine combination, was also demonstrated in vivo in CK-AML. We hypothesized that PegC-mediated plasma glutamine depletion inhibits 4EBP1 phosphorylation, decreases the expression of proteins such as MCL-1, whose translation is cap dependent, synergizing with the BCL-2 inhibitor Ven. Ven-PegC treatment decreased cellular MCL-1 protein levels in vitro by enhancing eIF4E-4EBP1 interaction on the cap-binding complex via glutamine depletion. In vivo, Ven-PegC treatment completely depleted plasma glutamine and asparagine and inhibited mRNA translation and cellular protein synthesis. Since this novel mechanistically-rationalized regimen combines two drugs already in use in acute leukemia treatment, we plan a clinical trial of the Ven-PegC combination in relapsed/refractory CK-AML.

## Introduction

Acute myeloid leukemia with complex karyotype (CK-AML), defined as harboring three or more unrelated chromosome abnormalities, comprises 10-12% of AML cases and is the second largest cytogenetic subset in patients with AML [1]. CK-AML has a dismal outcome with intensive chemotherapy, DNA methyltransferase inhibitor (DNMTI) therapy, as well as allogeneic hematopoietic stem cell transplantation [2, 3]. CK-AML also commonly (70–80%) has *TP53* mutations [4], which are also associated with dismal outcomes [5]. Improving outcomes for patients with CK-AML will require approaches involving novel drug combinations that target the vulnerabilities of CK myeloblasts, such as cellular protein translational machinery [6].

Venetoclax (Ven) is an orally bioavailable small molecule that specifically inhibits binding of BIM and BAX proteins to BCL-2, resulting in activation of the pro-apoptotic protein BAK, which triggers apoptosis via mitochondrial outer membrane permeabilization and activation of caspases [7]. In 2018, Ven, in combination with a DNMTI, azacitidine or decitabine, or low-dose cytarabine, was approved by the Food and Drug Administration (FDA) for the treatment of newly diagnosed AML in adults who are age 75 years or older, or who have comorbidities that preclude use of intensive induction chemotherapy [8, 9]. In this patient population, Ven in combination with a DNMTI resulted in ~60% complete remission with full or partial count recovery (CR + CRh) with median duration of response of 4.7–5.5 months [8]. While results of Ven in combination with DNMTIs for newly diagnosed AML patients are encouraging, Ven in combination with DNMTIs is less effective in patients with relapsed or refractory (R/R) AML [10, 11]. CK-AML is commonly refractory to initial therapy, and, if it responds, it relapses rapidly [12].

AML cells are sensitive to extracellular glutamine depletion or manipulation of intracellular glutamine metabolism [13, 14]. Asparaginase, a component of a multi-agent chemotherapeutic regimen for treatment of pediatric and adult patients with acute lymphoblastic leukemia (ALL), converts asparagine and glutamine to aspartate and glutamate, respectively, decreasing plasma concentrations of asparagine and glutamine [15]. Clinical asparaginases are isolated from either *E. coli* (e.g., pegaspargase) or *Erwinia chrysanthemi* (crisantaspase); the latter having higher glutaminase (i.e., glutamine decreasing) activity [16, 17]. In our previous clinical study, crisantaspase produced complete plasma glutamine depletion in patients, with no dose-limiting toxicity, and was associated with anti-leukemic activity in R/R AML [18]. Long-acting crisantaspase, Pegcrisantaspase (PegC), is a recombinant pegylated *Erwinia* asparaginase that has been used in pediatric patients with ALL [19].

Interference with glutamine metabolism has been shown to overcome resistance to BCL-2 inhibition in AML and other cell types [20-22]. We therefore hypothesized that depletion of glutamine induced by PegC would not only inhibit proliferation of CK-AML but also enhance the anti-apoptotic activity of Ven-mediated antagonism of BCL-2 in CK-AML by decreasing the expression of proteins such as MCL-1, whose translation is dependent on eukaryotic translation initiation factor 4E (eIF4E)- eIF4E-binding protein (4EBP1) interaction. Here, we report potent anti-leukemic activity of the Ven-PegC combination in CK-AML in vitro and in vivo. We further show that Ven-PegC blocks synthesis of proteins including MCL-1 in CK-AML cells by enhancing eIF4E-4EBP1 interaction on the cap-binding complex thereby inhibiting cap-dependent translation of mRNA.

## Results

### PegC has potent single-agent anti-AML activity and synergizes with Ven in vitro

The anti-leukemic activity of PegC, Ven, and their combination was tested in six human CK-AML cell lines (U937, MOLM-14, MV4-11, MonoMac6, HL60, K562) in vitro. Karyotype and mutational analyses were performed on each cell line, confirming complex karyotypes and demonstrating TP53 mutations in three (Supplementary Table S1). Treatment with single-agent PegC decreased in vitro proliferation of AML cell lines in a concentration-dependent manner, with IC_50_s ranging from 0.0001 to 0.049 international units per milliliter (IU/mL) (Supplementary Fig. S1), indicating potent single-agent activity even when compared with ALL cell lines [23]. Importantly, these concentrations are pharmacologically relevant, as they are readily achieved in patients [24]. Ven monotherapy showed modest activity against CK-AML cell lines, with the following IC_50_s: U937 = 14.5 ± 1.5 μM, MOLM-14 = 5.2 ± 0.2 μM, MonoMac6 = 7.4 ± 1.3 μM, HL60 > 50 μM, MV4-11 = 0.03 ± 0.01 μM, and K562 = 11.2 ± 0.1 μM.

**Fig. 1:**
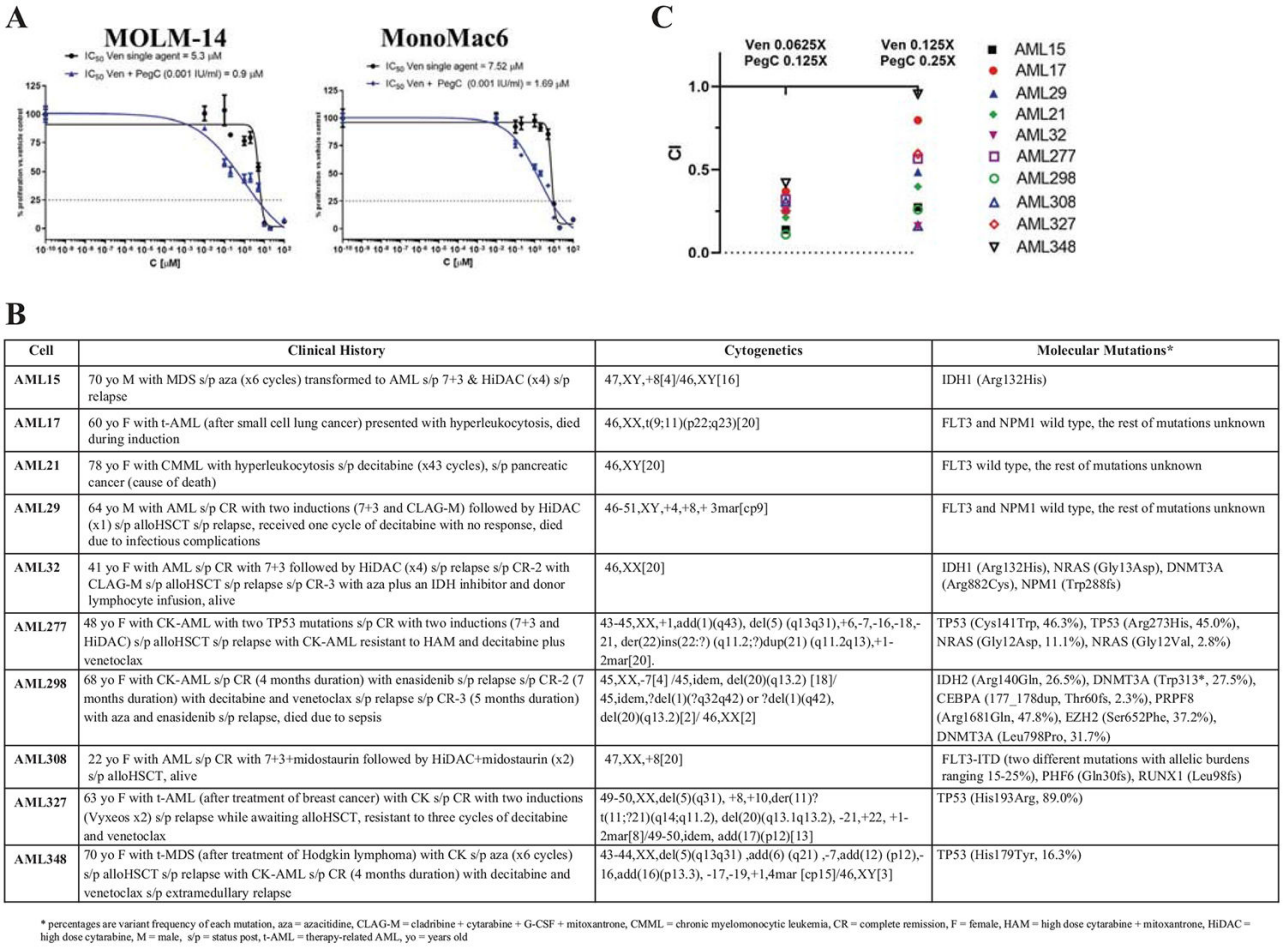
In vitro anti-AML activity of the combination of Ven and PegC. **a** PegC potentiates anti-AML effect of Ven in two human AML cell lines, MOLM-14 and MonoMac6. Cells were exposed for 72 hours (h) to Ven with vehicle (DMSO) or 0.001 IU/mL PegC. After WST-1 termination, IC_50_ values were calculated by GraphPad Prism. **b** Clinical course, cytogenetics and molecular mutations of patient-derived primary AML cells. **c** Primary AML cells were plated overnight then treated with fixed ratios of Ven and PegC (Ven 0.0625X = 1.5625 μM, 0.125X = 3.125 μM; PegC 0.125X = 0.003 IU/mL, 0.25X = 0.006 IU/mL). The cultures were terminated at 24 h and viability was assessed with alamarBlue. Combination Indices (CI) were calculated using Compusyn, free software utilizing Chou-Talalay’s method. CI values were plotted with their respective Ven-PegC combination ratios.

We then tested whether co-exposure to PegC at low concentrations could enhance the anti-leukemic activity of Ven. The clinically and regulatory relevant nadir serum asparaginase activity (NSAA) which can be achieved by asparaginase products is 0.1 IU/mL. Addition of PegC at 0.001 IU/mL (~1% of the clinical concentration) significantly increased the anti-AML potency of Ven, decreasing its IC_50_ 5–8 fold in MOLM-14 and MonoMac6 cells (Fig. 1a). Median effect analysis based on the Chou Talalay theorem was used to generate combination index (CI) values to determine if Ven and PegC synergize. A CI < 1 is synergistic, a CI = 1 is additive, and a CI > 1 is antagonistic. Treatment of MOLM-14 cells with Ven and PegC in fixed ratios showed synergistic (CI = 0.6) inhibition of leukemia growth by the two agents. Cell cycle analysis of MOLM-14 cells treated with Ven, PegC, and their combination showed decreased percentages of cells in S and G2 phase and increased cells in subG1 in combination-treated cells (Supplementary Fig. S2). Expression of the anti-apoptotic proteins BCL-2 and BCL-XL showed no alteration in MOLM-14 cells after treatment with Ven and/or PegC (Supplementary Fig. S3), suggesting that other mechanism(s) explain their synergistic cytotoxicity.

Next, we investigated the synergism between Ven and PegC against ten patient-derived primary AML cells including five CK-AML cells, three of which also had *TP53* mutations (Fig. 1b). To understand whether the approach may have broader applicability, we chose AML cells with various cytogenetics and mutational characteristics. Primary AML cells were exposed to Ven and PegC in fixed ratios for 24 h and viability was assessed with alamarBlue. Calculated CIs for all ten primary AML cells ranged from 0.11016 to 0.41967 at lower Ven and PegC concentrations, indicating synergistic inhibition of leukemia growth by Ven and PegC combination (Fig. 1c). Four CK-AML cells were derived from patients who were treated with combination of Ven and a DNMTI; two were resistant to Ven (AML277 and AML327) and two were sensitive (AML298 and AML348) with relatively short duration of response (≤5 months) (Fig. 1b).

The DNMTIs (or hypomethylating agents) azacitidine and decitabine are currently commonly used for treatment of patients with AML in both frontline and R/R settings. Addition of Ven or PegC to decitabine or azacitidine showed a modest additive effect or no effect, rather than synergism (Supplementary Fig. S4).

### Ven-PegC is well tolerated in vivo

After demonstrating in vitro efficacy of Ven-PegC, we next tested the combination in vivo. First, we tested the tolerability and safety of PegC monotherapy and Ven-PegC in non-leukemia bearing NRG (NOD.Cg-*Rag1^tm1Mom^ Il2rg^tm1Wjl^*/SzJ) mice. To determine the maximum tolerated dose (MTD) of PegC, we injected single-agent PegC intravenously (IV) once weekly for 2 weeks at 500 and 1000 IU/kg, which were higher doses compared with 5–250 IU/kg reported in the literature [25]. Mice lost ~20% body weight after these higher doses of single-agent PegC, and fatalities were observed (Supplementary Fig. S5A). The MTD for PegC monotherapy was determined to be 250 IU/kg (Supplementary Fig. S5B). To determine the MTD of the Ven-PegC combination, we treated NRG mice with 250 IU/kg PegC IV weekly plus a literature-based dosing schedule for Ven [26] of 100mg/kg PO 5 days per week. In response to significant weight loss (Supplementary Fig. S5B) after 1 week of treatment, we reduced the dose of Ven to 75 mg/kg and PegC to 200 IU/kg with no change in schedule.

We then tested the effect of Ven-PegC combination treatment for 2 weeks on complete blood counts (CBC), hepatic and renal function, pancreatic enzymes, and coagulation markers in immune-competent female and male CD1 mice. Weight loss was minimal and transient (Supplementary Fig. S5C). Following euthanasia after 2 weeks, whole blood and plasma were analyzed. Ven-PegC caused leukopenia, a known adverse event of Ven but not asparaginase (Supplementary Table S2), with no other blood cell count changes [8, 27]. Transaminases, bilirubin, amylase, and lipase were not elevated, indicating no hepatotoxicity or pancreatitis (Supplementary Table S2), nor were there substantial clinically relevant changes in fibrinogen or prothrombin time (PT) values (Supplementary Table S2). Similarly, the combination of Ven plus a different asparaginase (*E. coli* pegaspargase) caused no changes in blood cell counts or in a comprehensive metabolic panel (CMP) [28] (Supplementary Table S3).

### Combination of PegC and Ven demonstrates potent anti-AML efficacy in vivo

**Fig. 2:**
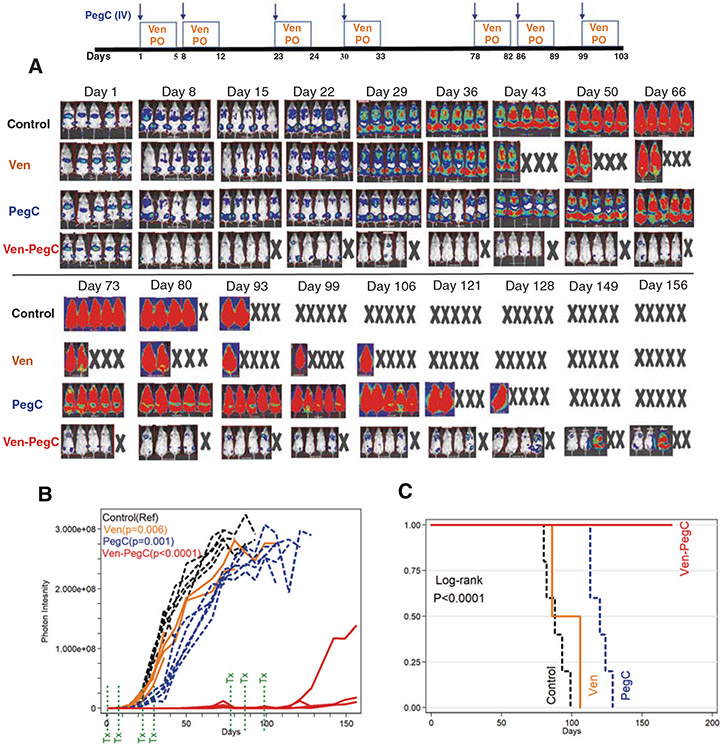
Efficacy of Ven, PegC and Ven-PegC combination in an orthotopic patient-derived xenograft (PDX) model of relapsed AML with complex karyotype. AML45-luc (1 × 10^6^ cells) were injected IV into NRG mice, and after confirmation of engraftment, mice were treated with vehicle, Ven, PegC, or Ven-PegC. Ven was dosed at 75 mg/Kg once daily on days 1–5, 8–12, 23–24, 30–33, 78–82, 86–89, and 99–103. PegC was dosed at 200 IU/Kg weekly on days 1, 8, 23, 30, 78, 87, 99. No treatment was administrated between Week 5 and Week 11. One mouse in the Ven-PegC group died in the second week due to a technical error. Mice were imaged weekly. **a** Imaging at serial time points. **b** Photon intensity vs. time. Photon intensity correlates with AML burden. Each line is a different mouse. The *p* values were calculated for each treatment groups compared with Vehicle. Ven-PegC combination was statistically significantly superior than Ven or PegC monotherapy. **c** Kaplan–Meier survival curve of mice treated with vehicle, Ven, PegC or their combination. Mice that died for reasons other than leukemia burden (Photon intensity < 1.5 × 10^8^) were censored. Ven-PegC compared with Vehicle, Ven or PegC improved overall survival of the mice.

To test the anti-AML efficacy of Ven-PegC combination, we developed a patient derived xenograft model (PDX) from primary cells (AML45) that were cryopreserved from a patient with relapsed CK-AML transformed from a myelodysplastic syndrome (MDS). We constructed the AML45-luc PDX model as luciferase-expressing cells. NRG mice transplanted with AML45-luc cells were imaged 3–10 days post transplant to confirm engraftment and assess AML45 burden, then groups were treated with vehicle, Ven, PegC or Ven-PegC (Fig. 2). Serial weekly imaging quantitation of AML burden demonstrated suppression of AML growth only in the Ven-PegC group. By Day 36, no leukemia was detected in mice treated with Ven-PegC, whereas all of the other mice had massive leukemia burden (Fig. 2a). Treatment was suspended during Days 36–77, and then reinitiated for a final 3 weeks (Days 78–103). Dosing was stopped at Day 36 because the mice receiving Ven-PegC had no detectable disease as measured by Bioluminescent imaging (Fig. 2a). By day 73, growth of leukemic disease was evident and dosing was re-initiated.

Quantitation of AML45-luc luminescence via in vivo imaging showed that while Ven-treated (*p* = 0.006) and PegC-treated (*p* = 0.001) groups of mice had slightly lower photon intensity (leukemia burdens) than the vehicle control group, mice in Ven-PegC group had substantially lower leukemia burdens (*p* < 0.0001) (Fig. 2b, Supplementary Fig. S6A). Only one mouse in the Ven-PegC group developed detectable AML45-luc luminescence after Day 100. Ven-PegC treated mice had transient early weight loss but recovered and gained weight. Notably, retreatment at week 11 caused no additional weight loss (Supplementary Fig. S6B). Overall survival results were consistent with tumor burden measurements, with mice treated with Ven-PegC living significantly longer than all other mice (log rank *p* < 0.0001) (Fig. 2c).

**Fig. 3:**
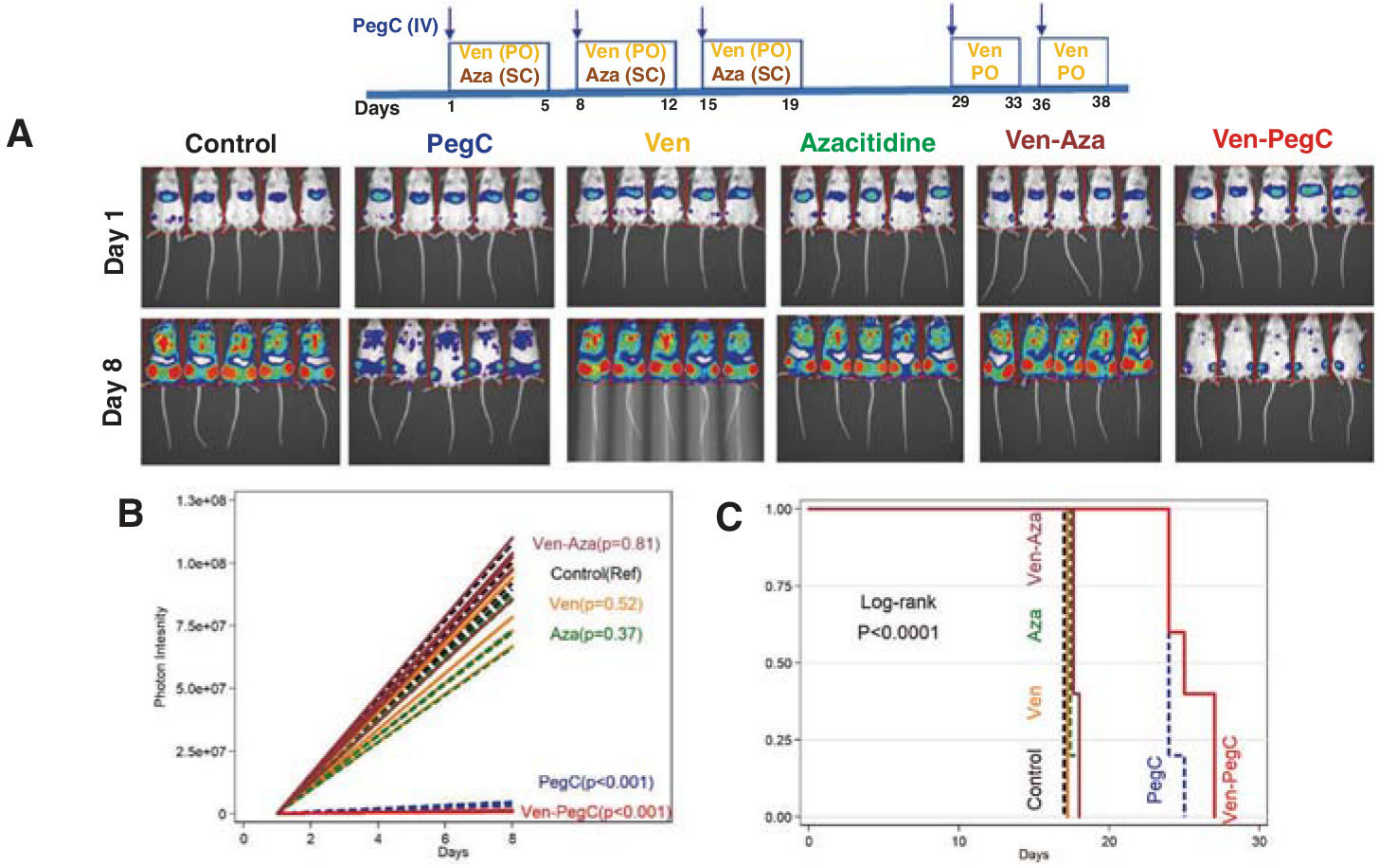
Efficacy of single agents Ven, PegC, azacitidine (Aza), and combination of Aza-Ven and Ven-PegC in U937-luc cells. U937-luc cells (0.25 × 10^6^) were injected IV into NRG mice. Following engraftment, mice were treated with PegC (200 IU/kg IV weekly) and/or Ven (75 mg/kg PO 5 days weekly) and/or azacitidine (0.5 mg/kg subcutaneously 5 days weekly). Mice were imaged weekly and survival was monitored. Ven was dosed on days 1–5, 8–10, PegC on days 1, 8, and azacitidine on days 2–5, 8–12. In the Ven-PegC group (the only mice to live beyond day 18), mice were treated with one more dose of PegC and 3 more doses of Ven. **a** Imaging at serial time points. **b** Photon intensity vs. time. Photon intensity correlates with AML burden. Each symbol represents a different mouse. **c** Kaplan–Meier survival curve of mice treated with vehicle, Ven, PegC or their combination, Aza and Aza-Ven.

The Ven-PegC combination was then evaluated in another CK-AML xenograft model, the U937 cells that showed in vitro sensitivity to PegC with a low IC_50_. U937 cells were chosen because they had the highest sensitivity to PegC of the AML cell lines evaluated (Supplementary Fig. S1). We developed the U937-luc orthotopic in vivo model. Post engraftment, U937-luc-bearing NRG mice were treated with vehicle, Ven, PegC, Ven-PegC, azacitidine (Aza), or the FDA-approved Ven-Aza combination. After 1 week of dosing, leukemia burden measured by U937-luc bioluminescence had increased markedly in the control, Ven, Aza, and Ven-Aza treated groups, but not the Ven-PegC-treated or, to a lesser extent, PegC-treated groups (Fig. 3a, b; *p* < 0.001 for Ven-PegC and PegC vs control, Supplementary Fig. S6C for Ven-PegC vs PegC). All mice in the vehicle-treated, Ven-treated, Aza-treated, and Ven-Aza-treated groups died by Day 18 (Fig. 3a-c), while survival was significantly prolonged in groups treated with Ven-PegC or PegC. The advantage of Ven-PegC over PegC in reducing leukemia burden with U937 cells was not quite as large as that seen for other cell lines. However, there was still a highly statistically significant fourfold difference in photon intensity between these two treatment arms, with photon intensities of 4.2 × 10^6^ in the PegC group, compared to 1.3 × 10^6^ in the Ven-PegC group (t-test *p* value < 0.01; Supplementary Fig. S6C).

### Ven-PegC alters transcription and inhibits p90RSK transcript in AML cells

**Fig. 4:**
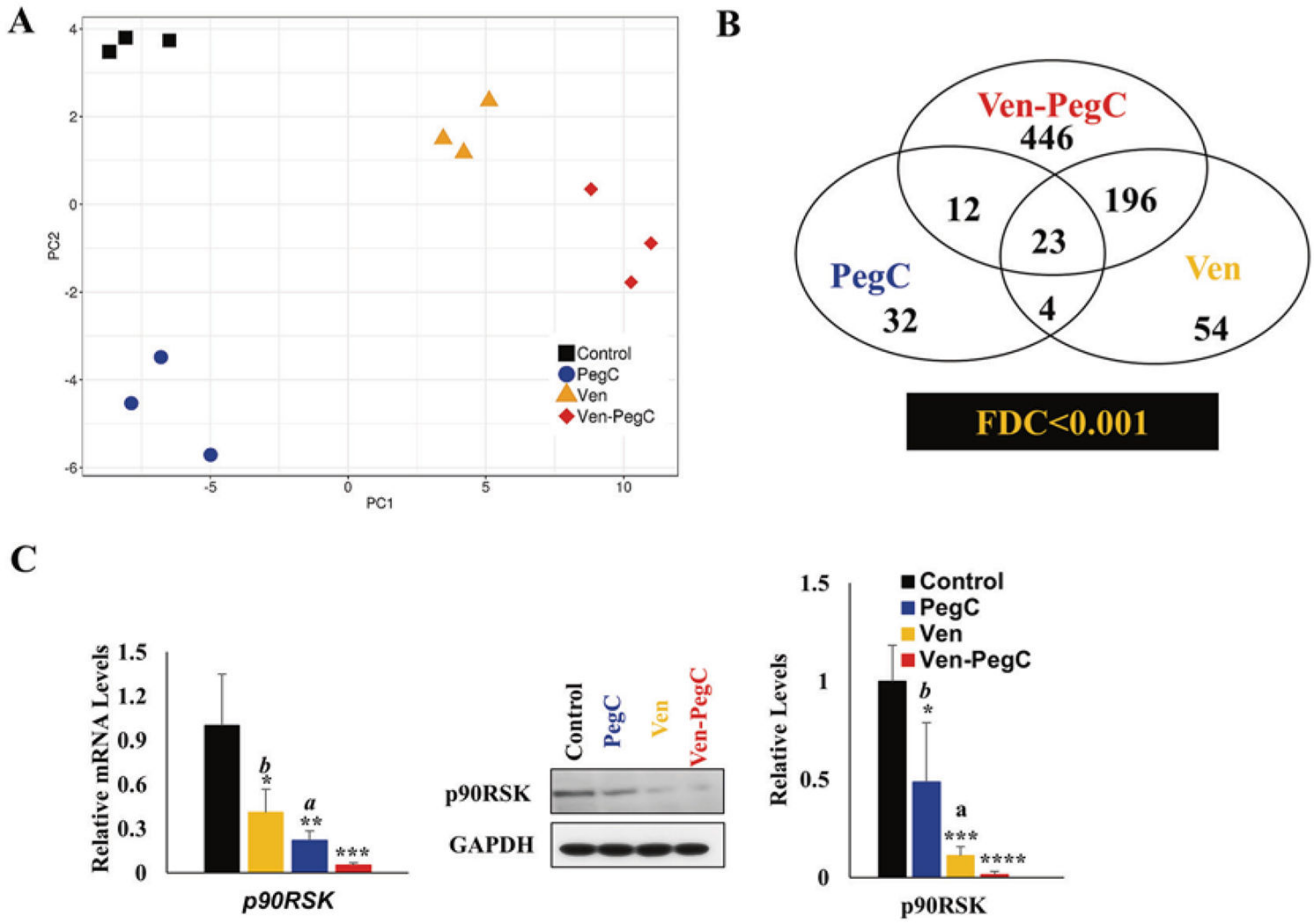
Alteration of gene transcription in AML after treatment with Ven, PegC, and Ven-PegC. **a** Principal component analysis (PCA) of whole-transcriptome RNA-seq data from the indicated treatment. **b** Venn diagram representing the number of differentially expressed genes (DEG) between Ven-, PegC-, and Ven-PegC-treated samples. The numbers and overlap regions in the Venn diagram show the variants unique to or shared among three individual treatment. Statistical analysis of the transcriptome at the fix dose concentration/combination (FDC) was significant (*p* < 0.001). **c** qRT-PCR analysis of p90RSK expression in MOLM-14 cells treated with control, Ven, PegC and Ven-PegC for 16 h (left) Western Blot analysis of p90RSK protein level in MOLM-14 cells treated for 16 with control, Ven, PegC and Ven-PegC. Results were normalized to control-treated cells and expressed as mean ± SD (*n* = 3) (right). Statistical analysis was performed using one-way ANOVA, and *p* values were adjusted using Bonferroni’s correction method *p < 0.05, **p < 0.01, ***p < 0.005 vs control-treated corresponding cells, ^a^*p* < 0.05, ^b^*p* < 0.01 vs Ven-PegC treated corresponding cells.

With the observed in vitro and in vivo efficacy results, we sought to investigate the mechanism of action of Ven-PegC combination. We hypothesized that glutaminase activity of PegC inhibits 4EBP1 phosphorylation and decreases the expression of proteins such as MCL-1, whose translation is cap dependent, synergizing with the BCL-2 inhibitor Ven. We first performed whole-transcriptome/gene expression profiling (GEP) in MOLM-14 cells treated with Ven, PegC, or Ven-PegC for 16 h using RNA sequencing (RNA-seq) (Supplementary Fig. S7A, S7B and Supplementary Table S4). Principal Component Analysis (PCA) of these transcriptomic data indicated distinct clusters for replicates in each treatment group and diverse clusters among different conditions (i.e., vehicle control, Ven, PegC, and Ven-PegC) on the basis of their GEP (Fig. 4a). RNA-Seq analysis identified more than 30,000 genes in each dataset with relatively high expression levels, covering more than 91% of exonic regions (Supplementary Table S4).

Using multivariable linear regression analysis, we observed significant gene expression changes following Ven (*n* = 277) and PegC (*n* = 71) monotherapy, as compared to vehicle control, as shown in the heatmap derived from transcriptome data analysis (Supplementary Fig. S7C, S7D and Supplementary Table S5 [as a separate Excel database]). A robust modification of mRNA levels (*n* = 677, 282 upregulated/395 downregulated) was noted after treatment with the Ven-PegC combination, as compared to vehicle (Supplementary Fig. S7D), which was more than the number of RNA transcripts modified with each individual agent alone (Supplementary Fig. S7D). These results corroborate our in vitro findings that Ven and PegC combination acts synergistically in AML cells.

While there was a low correlation of GEP for PegC vs Ven (R = 0.58, Supplementary Fig. S8A) and PegC vs Ven-PegC (R = 0.49, Supplementary Fig. S8B), there was a high correlation of GEP for Ven vs Ven-PegC (R = 0.85, Supplementary Fig. S8C). Treatment with Ven, PegC, or Ven-PegC altered expression of different genes (Supplementary Fig. S9). These genes were selected to delineate the specificity of each treatment, based on the transcriptome dataset as well as on their proposed impact on leukemia cell survival. Expression of genes namely, ASNS (Asparagine Synthetase) [29] and SLC6A9 (Solute Carrier Family 6 Member 9, Glycine transporter) [30] were altered with PegC treatment (and not Ven treatment), while the expression of oncogenes like HK3 (Hexokinase 3) [31] and DUSP4 (Dual specificity protein phosphatase 4) [32] were altered after Ven treatment (and not PegC treatment). Using the differentially expressed genes, we performed Kyoto Encyclopedia of Genes and Genomes (KEGG) pathway and Gene Ontology (GO) term enrichment analysis to assess the similarities and differences in molecular processes associated with each treatment. KEGG pathway hematopoietic cell lineage development was highly enriched in all treatments. Toll-like receptor (TLR) signaling pathway and transcriptional regulation in cancer were enriched in Ven and Ven-PegC treatment, while Janus kinases (JAK), signal transducer and activator of transcription proteins (STAT), mTOR and Interleukin 17 (IL17) were noted to be enriched upon PegC treatment. To assess the possibilities of altered molecular events upon compound treatment, we scanned the transcriptome profile using GO molecular functions. Based on transcriptome analysis, ribosomal activity/regulatory pathways (PI3K) along with TLR signaling pathways were enriched in all conditions. Amino acid transporter activity events were augmented upon PegC treatment while receptor for advanced glycation endproducts (RAGE) receptor binding was enhanced upon Ven as well as Ven-PegC treatment (Supplementary Table S6).

Next, we focused on the 23 candidate genes that were modulated by Ven and PegC and Ven-PegC (Fig. 4b and Supplementary Table S7). Among these 23 genes, we observed that p90 ribosomal S6 kinase 2 (p90RSK2, also known as RPS6KA2) levels were significantly decreased in cells treated for 16 h with PegC or Ven (*p* < 0.005) and robustly decreased with Ven-PegC (*p* < 0.001) (Fig. 4c). Western blot analysis after a 16 h treatment confirmed downregulation of p90RSK by Ven-PegC in MOLM-14 (Fig. 4c) and MonoMac6 cells (Supplementary Fig S10A). p90RSK2, which is hyperactivated in diverse myeloid leukemia cell lines, is an essential signaling kinase regulating cell proliferation and survival in myeloblasts carrying FMS-like tyrosine kinase 3-internal tandem duplication (FLT3-ITD) [33].

### Ven-PegC enhances 4EBP1/eIF4E interaction on cap-binding complex, inhibits cap-dependent translation, and decreases translational complex formation

**Fig. 5:**
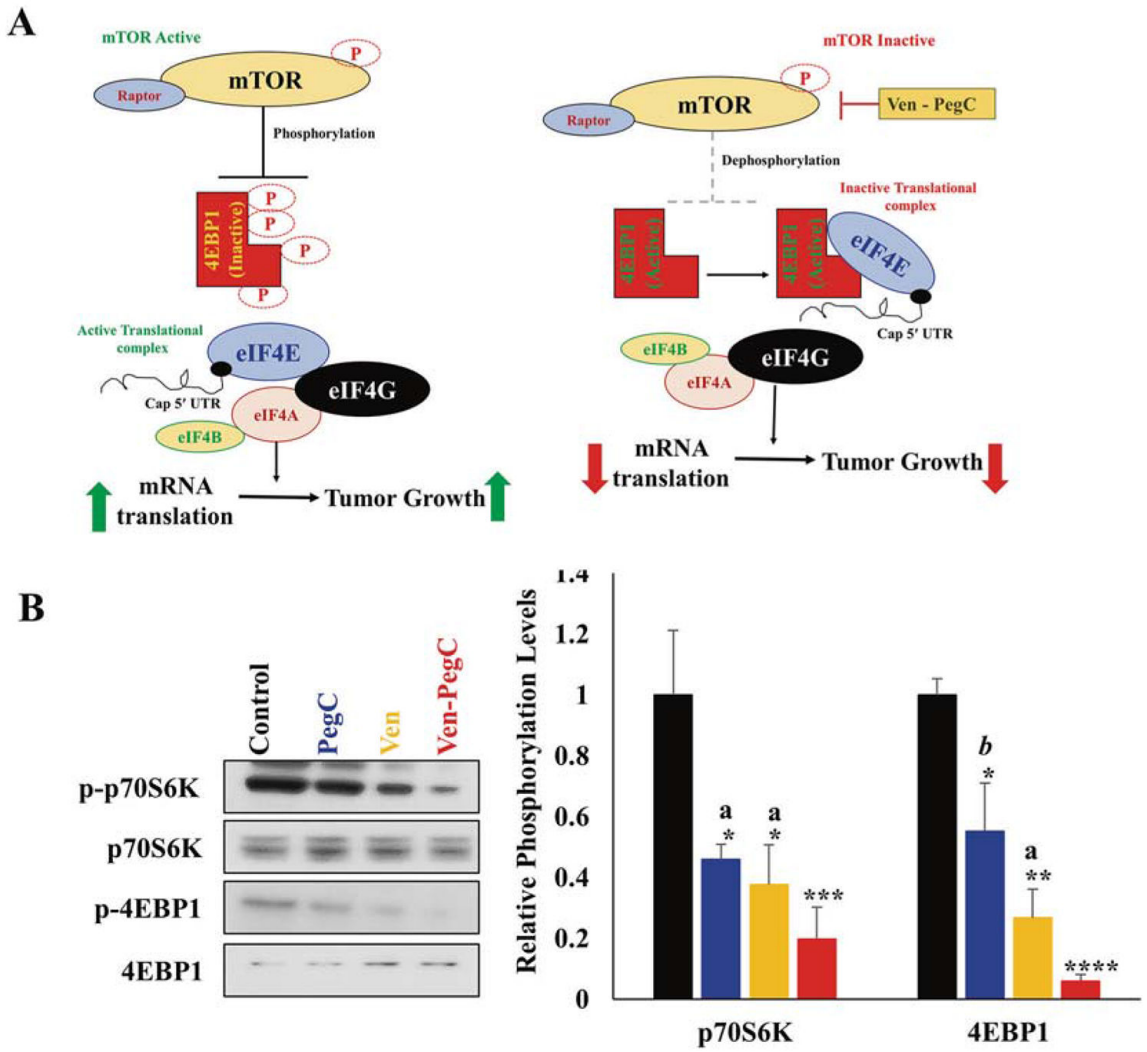
Ven-PegC impedes cap-dependent translation and protein synthesis. **a** Effect of Ven-PegC on mTOR and cap-dependent mRNA translation. **b** MOLM-14 cells were treated with PegC, Ven and Ven-PegC for 16 h and lysed, and cellular lysates were probed with the indicated antibodies. The bar diagram represents densitometric quantification of three independent experiments.

Glutamine depletion induced by L-asparaginases was reported to inhibit 4EBP1 (a tumor suppressor regulated by oncogenic mTOR pathway) phosphorylation at residue S65 and decrease protein synthesis in AML cell lines, with a fourfold greater effect with *Erwinia* asparaginase than with *E coli* asparaginase [13]. The mammalian target of rapamycin (mTOR) is a vital converging point of intra- and extra-cellular signals, regulating many fundamental cellular processes including mRNA translation and evidence indicates that mTOR dysregulation is deeply implicated in leukemogenesis [34]. PI3K/AKT/mTOR pathway is noted to be upregulated in Ven resistant cancer cells [35]. Having observed that (i) PegC, a long-acting *Erwinia chrysanthemi* asparaginase, regulates p90RSK2 expression, (ii) asparaginases negatively impact the mTOR/p70RSK1 (p70S6K) pathway [13], (iii) ribosomal S6 kinase activity seems altered from GO-molecular functions enrichment analysis in all three treatments, and (iv) enhancement of anti-AML activity of Ven by PegC, we hypothesized that co-treatment with Ven and PegC significantly diminishes cellular protein synthesis through interference with active cap-mRNA translation downstream of mTOR signaling (Fig. 5a). Consistent with our hypothesis, phosphorylation of p70S6K (p-p70S6K) as well as its substrate 4EBP1 (p-4EBP1) decreased upon treatment with Ven-PegC, significantly more than Ven or PegC alone (Fig. 5b). Similar results were observed in MonoMac6 cells (Supplementary Fig. S10B).

**Fig. 6:**
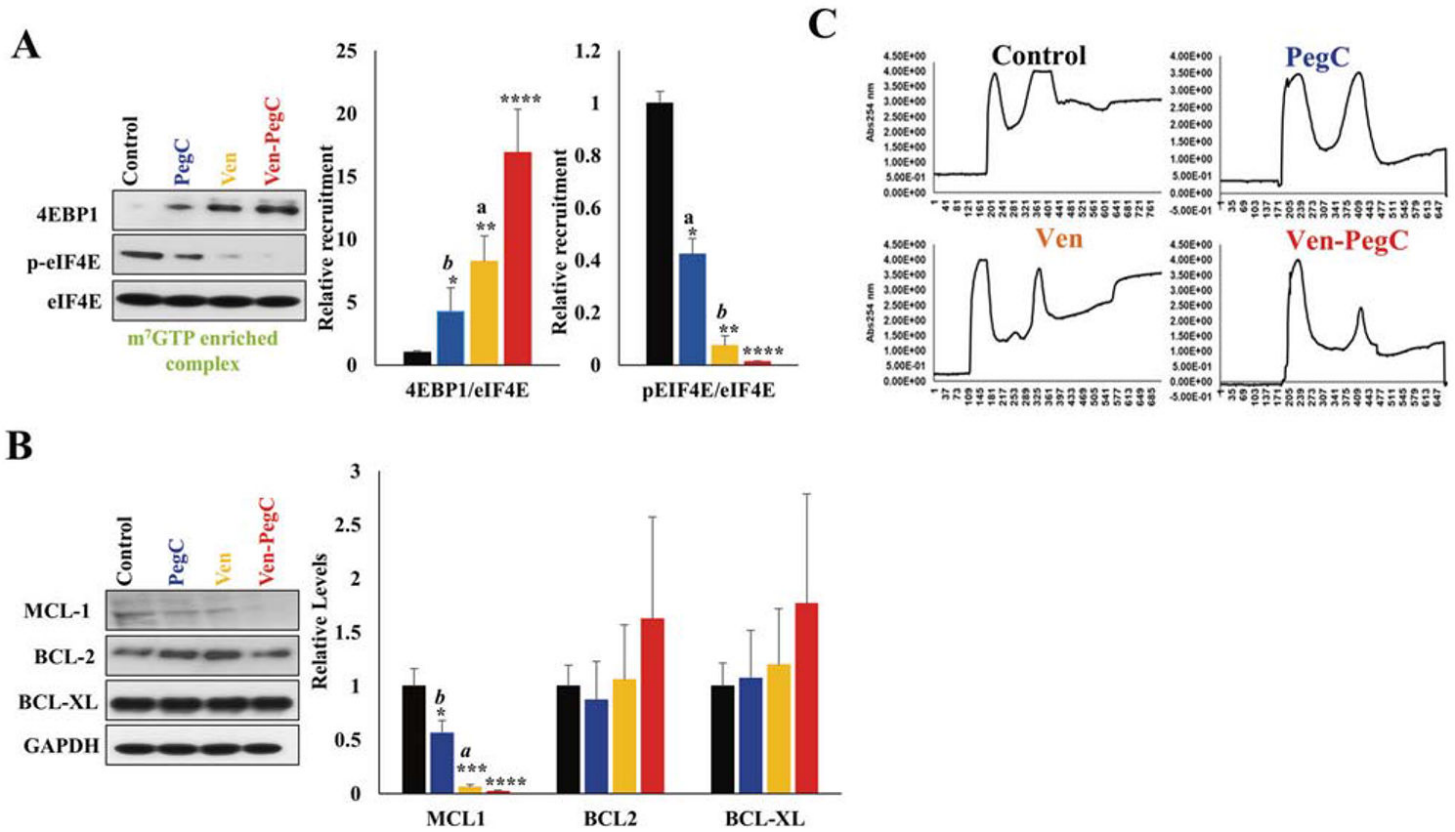
Ven-PegC impedes cap-dependent translation and protein synthesis. **a** Precleared cellular lysates of MOLM-14 cells treated with PegC, Ven and Ven-PegC were incubated with m^7^GTP sepharose beads for 2 h, followed by washing and probing with the indicated antibodies. The bar diagram represents densitometric quantification of three independent experiments. Results were normalized to corresponding control-treated cells and expressed as mean ± SD (*n* = 3). Statistical analysis was performed using one-way ANOVA, and *p* values were adjusted using Bonferroni’s correction method *p < 0.05, **p < 0.01, ***p < 0.005 vs corresponding control-treated cells, ^a^*p* < 0.05, ^b^*p* < 0.01, ^c^*p* < 0.005 vs corresponding Ven-PegC-treated cells. **b** MOLM-14 cells were treated with PegC, Ven and Ven-PegC for 16 h and lysed, and cellular lysates were probed with the indicated antibodies. The bar diagram represents densitometric quantification of three independent experiments. **c** Polysomal profiles of MOLM-14 cells treated with PegC (0.025 IU/mL) and/or Ven (5.2 μM) or DMSO control for 16 h. Areas under the translational initiation complex (80 S) were significantly reduced in all conditions compared to control. PegC polysomal capacity was significantly compromised compared to Ven and Control. Ven-PegC shows very minimal translational complex formation.

Next, we performed m^7^GTP enrichment experiments and probed for recruitment levels of 4EBP1 and pSer209-eIF4E (the active form of eIF4E) on the cap-binding complex. Cap-dependent mRNA translation is a key step in ribosomal output and its activity is reported to be augmented in a variety of cancers including leukemia [36]. We observed increased recruitment of 4EBP1 to cap complexes (Ven-PegC > Ven > PegC) (Fig. 6a). In contrast, a significant reduction in the phosphorylation of eIF4E was noted on the cap complex (Fig. 6a and Supplementary Fig. S10C). Moreover, in AML cell lines Ven-PegC significantly decreased protein levels of MCL-1 (but not BCL-2 or BCL-XL), whose translation is highly dependent on 4EBP1/eIF4E activity on mRNA-capping (Fig. 6b and Supplementary Fig. S10D) [37, 38].

These results indicate a strong tethering of 4EBP1 with eIF4E in formation of inactive translational initiation complexes, and a global reduction of cap-dependent protein translation by Ven-PegC, which then prompted us to perform ribosomal profiling to measure the formation of actively translating ribosomes. After treatment of MOLM-14 cells with PegC and/or Ven for 16 h, ribosomal fractions were enriched by sucrose gradient followed by RNA isolation. The area under the translation initiation complex (80S) decreased with all drug treatments, as compared to vehicle (Fig. 6c). Polysomal capacity decreased significantly with PegC treatment, compared to Ven and control, while very minimal translational complex formation was observed with Ven-PegC treatment, indicating a robust reduction of protein translation (Fig. 6c).

### PegC effectively depletes plasma glutamine and modulates protein translation-associated molecules in vivo

**Fig. 7:**
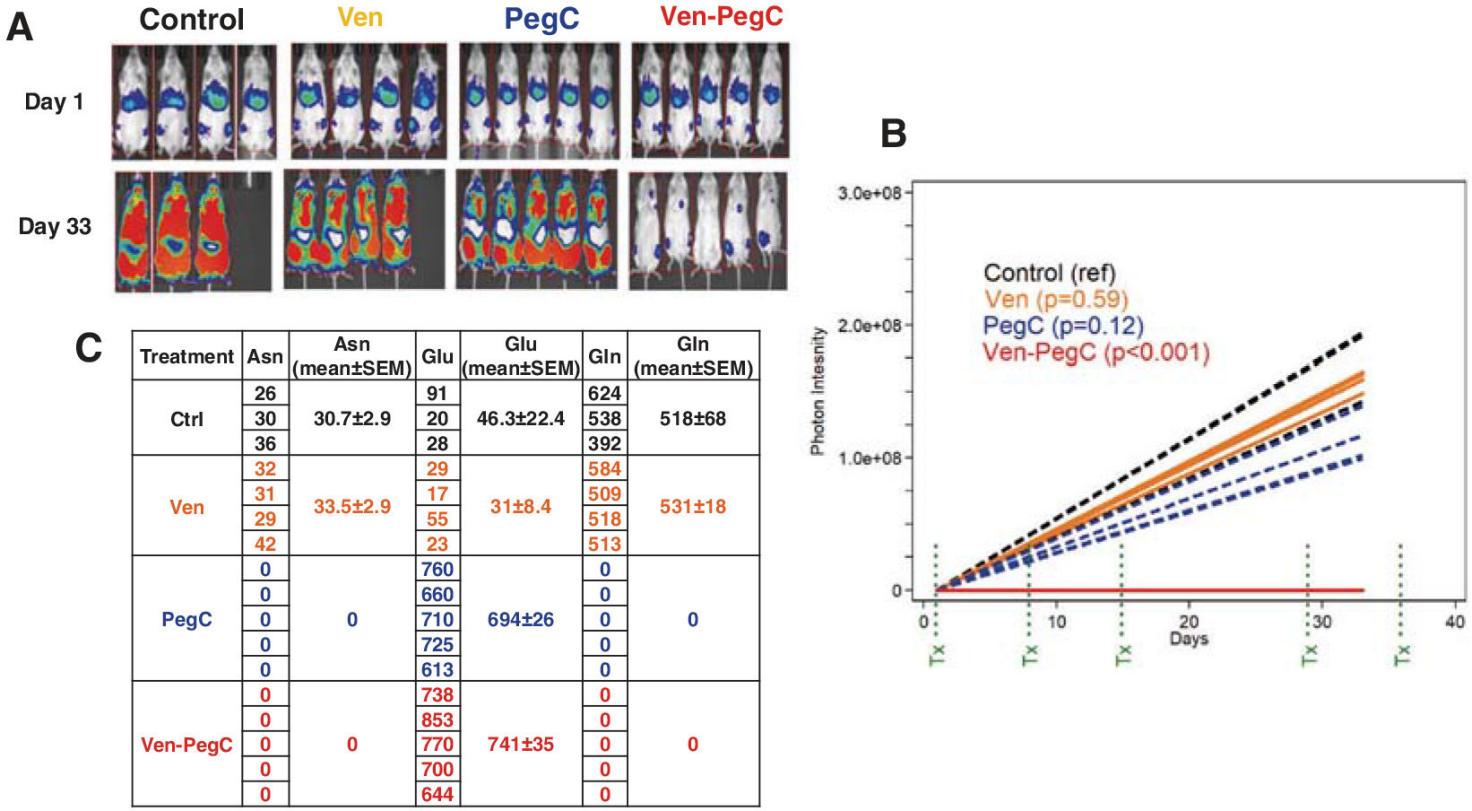
Effect of Ven-PegC on plasma amino acid levels in vivo in AML45-luc. AML45-luc (1 × 10^6^) cells were injected IV into NRG mice. After confirmation of engraftment by imaging, mice were treated with vehicle, Ven, PegC or Ven-PegC. Mice were dosed with PegC on days 1, 8, 15, 29 and 36, and Ven on days 1-5, 8–12, 15-9, 29-33 and 36-38. Mice were imaged on day 33 and euthanized on day 39. **a** Imaging on Days 1 and 33. **b** Photon intensity vs. time. Each line represents a different mouse. **c** Gln, Asn, and Glu levels (nmol/mL) levels in plasma from mice euthanized post last dose of treatments.

To confirm the observed in vitro effect of Ven-PegC on cap-dependent protein translation as well as its anti-AML efficacy in vivo; we performed a third in vivo PDX experiment with pharmacodynamic (PD) studies. We treated another cohort of mice engrafted with AML45-luc cells with vehicle, Ven, PegC, or Ven-PegC and terminated the experiment on Day 39 in all animals to collect blood for plasma amino acid analysis and bone marrow for immunoblotting and qRT-PCR analyses. Ven-PegC again showed clear superiority to the other treatments (Fig. 7a, b).

Our in vivo PD studies focused on comparing plasma concentrations of glutamine, asparagine, and glutamate between 10 mice that did and 7 mice that did not receive PegC using Mann—Whitney *U* tests. Plasma concentrations of glutamine and asparagine were undetectable in the 10 mice treated with PegC or Ven-PegC, compared with 525.4 and 32.3 micromolar (μM), respectively, in the 7 mice not treated with PegC (*p* = 0.0001 for both comparisons) (Fig. 7c). As expected, plasma glutamate increased more than 20-fold in mice that received PegC (713 μM vs. μM 38, *p* = 0.0006) (Fig. 7c), as the glutaminase activity of PegC converts glutamine to glutamate and ammonia. No significant differences in amino acid levels were noted in Ven-treated mice.

**Fig. 8:**
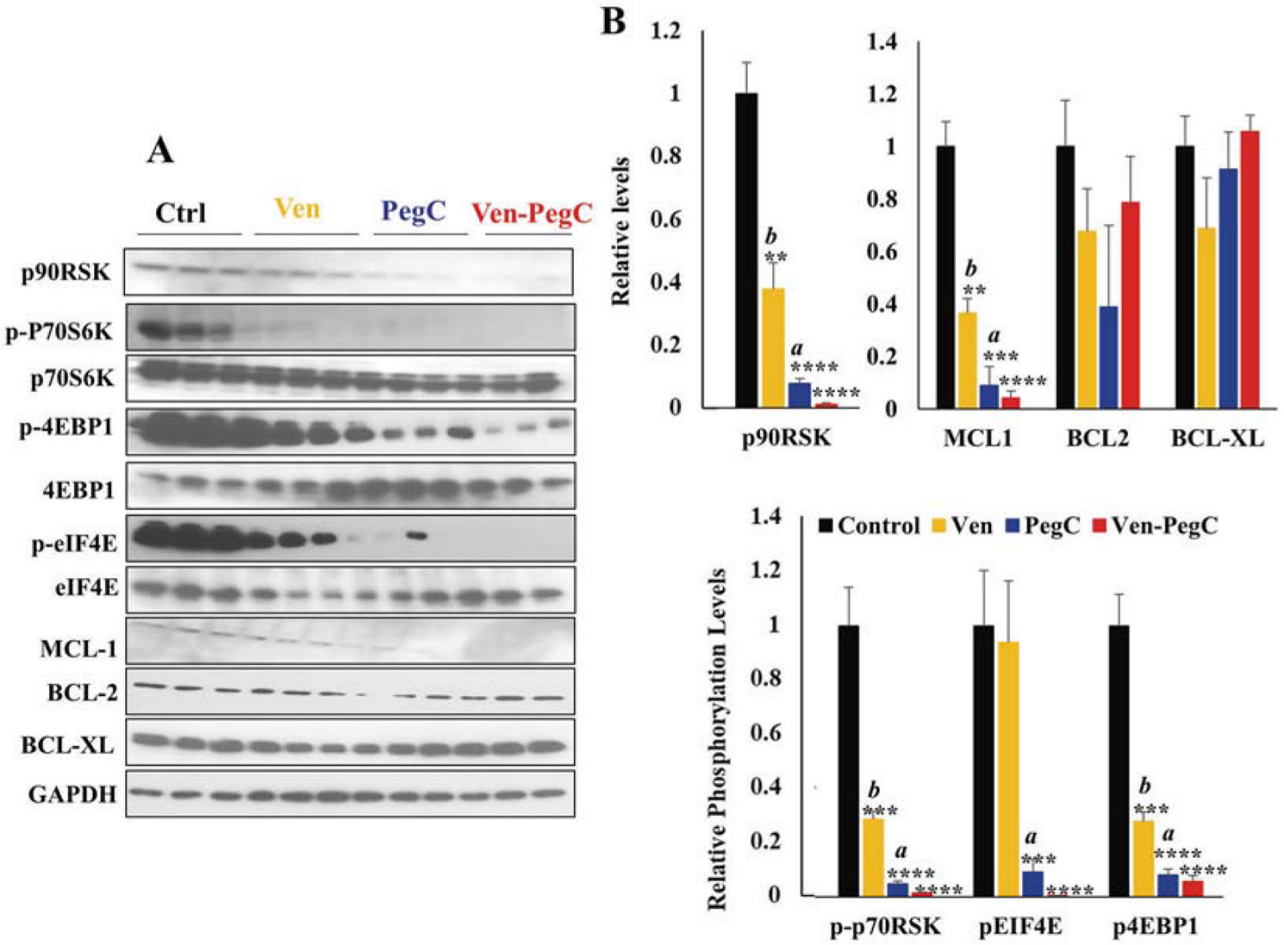
Pharmacodynamic (PD) effects of Ven-PegC in vivo in AML45-luc. **a** Western blot analysis of bone marrow lysates from mice treated with vehicle, Ven, PegC and Ven-PegC. **b** Bar diagram represents the densitometry quantification for three independent experiments. Results were normalized with Control-treated corresponding cells and expressed as mean ± SD (*n* = 3). Statistical analysis was performed using one-way ANOVA, and *p* values were adjusted using Bonferroni’s correction method *p < 0.05, **p < 0.01, ***p < 0.005 vs Control treated corresponding cells, ^a^*p* < 0.05, ^b^*p* < 0.01, ^c^*p* < 0.005 vs Ven-PegC treated corresponding cells.

To test the PD effect of in vivo Ven-PegC treatment on the expression of proteins identified in our mechanistic studies, protein lysates prepared from bone marrow mononuclear cells isolated from mice post-treatment were studied by western blot analysis. Consistent with our in vitro results on molecules involved in the translational phase of protein synthesis, we observed significantly decreased p90RSK expression and phosphorylation of p70S6K, 4EBP1, and eIF4E in bone marrow cells of Ven-PegC-treated mice (Fig. 8a, b). In addition, protein expression of MCL-1, but not BCL2 or BCL-XL, was decreased substantially in Ven-PegC-treated mice (Fig. 8a, b). mRNA expression of the genes modulated after PegC, Ven and Ven-PegC treatment in PDX mice was consistent with the in vitro transcriptome profile (Supplementary Fig. S11). These PD results confirm effective inhibition of mRNA translation and cellular protein synthesis as the anti-leukemic mechanism of action of Ven-PegC in vivo.

## Discussion

Here we show efficacy and the synergistic mechanism of the novel combination of PegC and Ven in CK-AML cell lines and primary cells in vitro and in vivo.

Although Ven combined with DNMTIs is effective as frontline AML therapy, single-agent Ven resulted in only ~20% overall response rate, with a median time to AML progression of <3 months [39]. In the relapsed setting, treatment with Ven in combination with DNMTIs resulted in an equally poor ~20% overall objective response rate in AML, with only half of responses being complete remissions with or without count recovery [11]. A major cause of poor response to Ven is overexpression of other anti-apoptotic proteins [40, 41]. Among these proteins, MCL-1 overexpression has a key role in the mechanism of resistance to BCL-2 inhibition in general [42, 43] and to Ven in particular [44]. Hence, development of combination therapies including Ven and an agent with a novel mechanism of action that can directly or indirectly inhibit MCL-1 and overcome Ven resistance is required. While first-in-human clinical trials of single-agent direct MCL-1 inhibitors (e.g., NCT03465540, NCT02675452, NCT03672695) are ongoing, the adverse event profile of these agents, including cardiotoxicity, may be prohibitive. In addition, combination of BCL-2 and MCL-1 inhibitors seems to have substantial toxicity to normal tissues including hematopoietic stem cells [45], hepatocytes [46] and cardiomyocytes [47]. Therefore, indirect induction of MCL-1 downregulation might be a practical strategy to circumvent Ven resistance in the clinic. MCL-1 protein has a short half-life, and maintenance of cellular MCL-1 protein levels is dependent on active cap-mRNA translation [48].

Assembly of the translation initiation complex eIF4F on the 5′ cap structure, followed by recruitment of ribosomal subunits is necessary for most mRNA translation and protein synthesis [49]. The eIF4E participates in the eIF4F complex, and its activity is regulated by mTOR through phosphorylation of the 4EBP1. mTOR-mediated phosphorylation of 4EBP1 releases eIF4E permitting it to enter the eIF4F complex. eIF4E is considered an oncogene and is overexpressed in several tumors [48]. One target of phosphorylated eIF4E is MCL-1 and augmented translation of MCL-1 probably contributes to eIF4E-mediated oncogenesis [50]. Downregulation of MCL-1 via inhibition of translation, but not decreased protein stability or increased ubiquitination, was reported to be the main mechanism of apoptosis in human leukemia cells [51]. Asparaginase-induced inhibition of 4EBP1 phosphorylation, necessary for maintenance of active cap-mRNA translation via release of eIF4E, has been reported to decrease MCL-1 expression [13].

We hypothesized that PegC-mediated plasma glutamine depletion might synergize with Ven against AMLs via downregulation of synthesis of cellular proteins including MCL-1. We have shown that short-acting *Erwinia* asparaginase, also called crisantaspase, can completely deplete plasma glutamine in patients with R/R AML and can provide clinical benefit [18]. Our current pre-clinical study focused on combining long-acting crisantaspase, PegC, with Ven at clinically relevant concentrations. The most significant result of our global transcriptome profile was modulation of the ribosomal biogenesis pathway. Together, Ven-PegC diminished p90RSK expression and phosphorylation of p70S6K, augmented eIF4E-4EBP1 interaction on cap-binding complexes, and resulted in inhibition of cap-dependent translation of proteins including MCL-1. This strong interference with cellular protein synthesis resulted in effective cell killing in vitro in CK-AML [52].

In order to test the combination of Ven-PegC in vivo, we focused on a PDX model of CK-AML, one of the most dismal subtypes of AML. Leukemia relapse is currently the most common cause of treatment failure and mortality for patients with CK-AML even after stem cell transplantation [3]. In our in vivo efficacy experiment, we estimated and compared survival by study arm using Kaplan–Meier curves and log-rank tests. Mice died due to leukemia when photon intensity reached a critical level of 1.5 × 10^8^. In our main in vivo study, we treated mice after confirmation of leukemia engraftment and carried out experiments for ~6 months, replicating real-world patient disease. All mice in the control, Ven, and PegC arms died due to leukemia within 130 days, while none of the mice in the Ven-PegC arm died due to leukemia during the study period (*p* < 0.0001). The superior efficacy of Ven-PegC was confirmed in a second cohort of mice with AML45-luc as well as in another in vivo model (U937-luc) of human CK-AML. Of note, AML is an extremely heterogeneous cancer with multiple subtypes, and there is a heterogeneity within the complex karyotype subcategory [53]. While we tested many AML cells with complex karyotypes in vitro and in vivo, the clinical response to Ven-PegC might differ depending on the number of aberrant chromosomes, presence of monosomy karyotype, or presence of *TP53* mutation.

In addition to being efficacious, Ven-PegC is also well tolerated. Notably, only one mouse in the Ven-PegC arm fell below 80% of its initial weight for ~10 days. All mice gained weight over time. Ven-PegC did not show any negative effect on organ function including asparaginase-related adverse events of special interest (i.e., elevated liver or pancreas enzymes, hyperbilirubinemia, coagulation tests and fibrinogen level) in immunocompromised and immunocompetent mice.

Our PD studies showed that PegC administration completely depleted plasma glutamine and asparagine, which is consistent with our Phase 1 clinical trial of short acting crisantaspase in patients with R/R AML. Measurement of proteins related to cap-dependent protein translation in the bone marrow cells of Ven-PegC-treated mice showed results consistent with the in vitro findings.

These promising pre-clinical data have prompted us to design a Phase 1 clinical trial of Ven-PegC for treatment of adult subjects with R/R AML including AML with complex karyotype and/or *TP53* mutation.

In conclusion, while BCL-2 inhibition with Ven is now commonly used for AML patients, our proposed combination of Ven and PegC is a novel, mechanistically-based regimen that is unique to the field and has the advantage of combining two drugs already in clinical use for acute leukemias, with use and potential toxicities familiar to oncologists.

## Materials and methods

### Cell Lines and Culturing

The human AML cell lines MOLM-14 and MonoMac6 were a kind gift from Dr. Mark Levis, Johns Hopkins University. MV4-11, U937, HL60 and K562 cells were purchased from ATCC (Manassas VA). Primary human leukemia cells were obtained through an IRB approved institutional tissue procurement protocol at the University of Maryland. Briefly, whole blood was received in sodium ethylenediaminetetraacetic acid (EDTA) tubes, and diluted 1:1 with phosphate buffered saline (PBS). Cells were isolated from diluted whole blood by Ficoll separation in lymphocyte separation medium (Corning Cellgro, Manassas, VA) spun at 400 × *g* for 30 min with no brake. Viable cell numbers were obtained using trypan blue exclusion. All cell lines were grown at 37°C with 5% CO_2_ atmosphere in Roswell Park Memorial Institute (RPMI) 1640 medium (Life technologies, Carlsbad, CA) supplemented with heat-inactivated 10% (V/V) fetal bovine serum (FBS) and 1% Glutamax (ThermoFisher Waltham, MA). Cell lines were grown and maintained according to ATCC recommendations. Primary cells were cultured in X-Vivo 15 Serum-free Hematopoietic cell medium (Lonza, Basel, Switzerland) supplemented with granulocyte-macrophage colony-stimulating factor (GM-CSF, Cell Signaling Technology, Danvers, MA) at 10 ng/mL, interleukin 3 (IL3, Cell Signaling Technology) at 50 ng/mL, recombinant human thrombopoietin (TPO, Biolegend, San Diego, CA) at 50 ng/mL, stem cell factor (SCF, Cell Signaling Technology) at 25 ng/mL, FLT3 ligand (FLT3L, Peprotech, Rocky Hill, NJ) at 50 ng/mL, interleukin 6 (IL6, Cell Signaling Technology) at 10 ng/mL, and granulocyte colony stimulating factor (Cell Signaling Technology) at 1 ng/mL. All cells were tested for mycoplasma contamination and utilized before 10 passages and treated in exponential growth phase at ~70% confluence. Human cell line authentication was confirmed using short tandem repeat analysis (STR) from the Gene-Print 10 STR typing kit^™^ (Promega Corp.) (Genomics Core at University of Maryland School of Medicine).

### AML Cells Karyotyping and Mutational Analysis

The assay was performed according to the manufacturer’s protocols, except that the GenePrint 5X mouse primer pair mix was added to the reaction to ensure that no mouse DNA contamination was present. Reactions were run on an Applied Biosystems model 3730XL sequencer and a 50 cm array, using GeneMapper software to collect and analyze data. Data were compared to various databases containing STR data for numerous cell lines, including ATCC and Cellosaurus.

*FLT3* Fragment Size Analysis was performed for the two common variants in patients with AML: *FLT3* internal tandem duplication (ITD) and *FLT3* tyrosine kinase domain (TKD) variant at D835. DNA from each cell line was independently amplified by polymerase chain reaction (PCR), using a fluorescently labeled dye attached to the forward primer sequence for each variant. *FLT3*-ITD was detected as a shift in mobility through a sequencing capillary, as detected on an Applied Biosystems model 3730XL. Using GeneMapper software, the size of the ITD and frequency were determined. Subsequent to PCR amplification, the D835 variant was identified by the ability of the restriction enzyme EcoRV to digest the PCR fragment, as the mutation occurs within an EcoRV restriction site. Using GeneMapper software and running through a sequencing capillary on the Applied Biosystems model 3730XL, identification of undigested PCR product was indicative of presence of a D835 variant and the frequency was calculated.

Next-generation DNA Sequencing for 31 genes associated with myeloid malignancies (*ASXL1, CALR, CBL, CEBPA, CSF3R, DNMT3A, ETNK1, EZH2, FLT3, GATA1, GATA2, IDH1, IDH2, JAK2, KIT, KMT2A, KRAS, MPL, NOTCH1, NPM1, NRAS, PTPN11, RUNX1, SETBP1, SF3B1, SRSF2, TET2, TP53, U2AF1, WT1, ZRSR2*) was performed using Ion Torrent technology. Using the Ion Torrent Ion Chef, we followed protocols developed by the manufacturer for preparing libraries, templating, and chip loading. Loaded chips were transferred to the QuantStudio S5 for sequencing on an Ion Torrent 520 chip, following the manufacturer’s protocols. After initial data analysis by the S5 software, data were analyzed using Ion Reporter software to identify variants of interest.

### Reagents and Chemotherapeutics

For in vitro studies, PegC was provided by Jazz Pharmaceuticals. Ven was purchased from LC Labs (Woburn, MA) as powder and dissolved in DMSO in 50–100 mM stock solutions and stored at −20 °C.

For in vivo studies, PegC was supplied by Jazz Pharmaceuticals at 500 IU/mL and stored at 4 °C. It was diluted in sterile PBS to the appropriate dosing solution concentration (e.g., 200 IU/kg = 20 IU/mL). Ven was dissolved in DMSO at 75 mg/mL, aliquoted and stored at −20 °C. It was then formulated fresh on the day of dosing as 10% DMSO, 30% PEG400, 60% Phosal PG50. Aza was purchased from Sigma Aldrich (Saint Louis, MO) and solubilized in sterile saline at 0.05 mg/mL and frozen at −80 °C. Aliquots were thawed daily and used immediately. For Reagents, see Supplementary Table S8.

### Cell Proliferation Assay

Cell lines and primary cells were seeded into 96-well plates the afternoon prior to treatment. Approximately 18h later, Ven and Peg-C serially diluted in vehicle or growth medium and added to cells. Plates were incubated for 72 h for cell lines and 48 h for primary cells prior to addition of water-soluble tetrazolium (WST-1) (Clontech, Mountain View, CA). Plates were read after 4 additional hours of incubation at 37 °C using a BioTek Synergy HT plate reader (BioTek, Winooski, VT). Data were analyzed and graphed using GraphPad Prism Software (Graphpad, La Jolla, CA) and IC_50_ concentrations were calculated.

### Combination Index

The effect of PegC in potentiating the cytotoxicity of Ven was investigated by conducting the proliferation assay with Ven at its IC_50_ concentration in the presence of PegC at its IC_10_ concentration. Agents were added simultaneously and cultures were terminated after 72 h followed by assessment of proliferation with WST-1. IC_50_s for the combined agents were calculated by GraphPad Prism. For synergism, both agents were added in fixed ratios (e.g., ¼xIC_50_, ½xIC_50_, 1xIC_50_, 2xIC_50_, 4xIC_50_) for 72 h followed by the addition of WST-1. Primary AML sample cultures were terminated at 24 h followed by the addition of alamarBlue. The optical density units from the WST-1 assay and the relative fluorescence units from the alamarBlue assay were analyzed by median effect analysis using Compusyn software (free online software based on the Chou Talalay theorem) [54]. Combination Index (CI) was generated; CI < 1 synergistic, CI = 1 additive, CI > 1 antagonistic.

### Cell Cycle Analysis

MOLM-14 cells were plated overnight then treated with vehicle, Ven (5.2 μM), PegC (0.025 IU/mL), or Ven-PegC combination. After 48 h, cells were fixed and permeabilized in ice-cold 70% ethanol for 2 h at −20°C then washed with cold PBS. Cells were resuspended in staining buffer (PBS with 0.5% BSA and 2 mM EDTA) with RNAase (100 μg/mL, Sigma) and propidium iodide (BioLegend, San Diego, CA) and incubated for at least 1 h at 4°C. Samples were run on the BD FACS Canto II and analyzed using FCS Express V6 (De Novo Software, Pasadena, CA).

### Western Blot Analysis

Cells were lysed with radioimmunoprecipitation assay (RIPA) buffer (Thermo Fisher Scientific) supplemented with cOmplete^™^, EDTA-free Protease Inhibitor Cocktail (Sigma Aldrich). After thorough mixing and incubation at 4°C for 30 min, lysates were centrifuged at 10,000 g at 4°C for 10 min, and supernatants were collected. Protein content of lysates was determined, and lysates were separated by 4–12% polyacrylamide gel electrophoresis (SDS-PAGE), and electro-transferred onto polyvinylidene difluoride (PVDF) membranes. After blocking with 5% non-fat milk in tris-buffered saline, 0.1% Tween 20 (TBST), membranes were incubated with primary antibodies at 4°C overnight, followed by 1:3000 horseradish peroxidase (HRP)-conjugated secondary antibody (Santacruz Biotechnology, Dallas, TX) for 1 h. Bands were visualized using Pierce Enhanced Chemiluminescence (ECL) Western Blotting Substrate (Thermo Fisher Scientific, Waltham, MA). Densitometric analyses were performed using Image Studio (Licor Biosciences) and presented as ratio of target band signal intensity to actin/GAPDH band signal intensity.

### Analysis of m^7^GTP-sepharose-bound proteins

The affinity purification of proteins associated with the m^7^GTP Sepharose (Jena Biosciences, Germany) was performed similarly to that described earlier [38]. Cell lysates were prepared after 24 h of treatment and incubated with m^7^GTP-sepharose for 2 h in cap-binding buffer (40 mM 4-(2-hydroxyethyl)-1-piperazineethanesulfonic acid [HEPES], pH 7.6; 120 mM NaCl; 1 mM EDTA; 0.3% 3-[(3-cholamidopropyl)dimethylammonio]-1-propanesulfonate. [CHAPS]). The beads were washed at room temperature three times in cap-binding buffer and boiled with 2X loading dye, followed by separation on 4–12% SDS-PAGE and probing with specific antibodies.

### RT-PCR

Total RNA was extracted using Tri Reagent (Sigma Aldrich) following the manufacturer’s protocol. 2 μg of total RNA was treated with DNase I (NEB, Ipswich, MA) and reverse transcribed into cDNA using High-Capacity cDNA Reverse Transcription Kit (Thermo Fisher), followed by qPCR with Power SYBR green PCR master mix (Thermo Fisher) on a QuantStudio 7 Flex Real-Time PCR System (Thermo Fisher). Primer sequences are available upon request.

### Polysomal fractionation

Polysomal fractionation was performed as previously reported [38]. Briefly 100 million cells were lysed in polysomal lysis buffer and fractionated using a linear sucrose gradient (10-50%). RNA was isolated and cDNA was prepared as mentioned earlier. RNA isolated from polysomal fractions was mixed in equal ratios and was utilized for RNA-Seq.

### Transcriptome Bioinformatic Analyses

The transcriptome samples were sequenced at the Institute for Genome Sciences (IGS), Baltimore, MD using the Illumina HiSeq sequencing platform. Raw sequencing reads generated for each sample were analyzed using the CAVERN analysis pipeline [55]. Read quality was assessed using the FastQC toolkit [56] to ensure good quality reads for downstream analyses. Reads were first aligned to the human reference genome build GRCh38 using HISAT2, a splice-aware alignment software for mapping next-generation sequencing reads [57]. Reads were aligned using default parameters and the strand-specific protocol to generate the alignment BAM files. Read alignments were assessed to compute gene expression counts for each gene using the HTSeq count tool [58] and the human reference annotation (GRCh38.91). The raw read counts were normalized for library size and utilized to assess differential gene expression between the control and drug-treated groups using the R package “DESeq” [59]. *P* values were generated using a modified Fisher’s exact test implemented in DESeq and then corrected for multiple hypothesis testing using the Benjamini–Hochberg correction method. Genes significantly differentially expressed between conditions were determined using a false discovery rate (FDR) of 5% and a minimum fold-change of 2X.

### In vivo tolerability and safety

For all studies, mice were housed under pathogen-free conditions in a University of Maryland Baltimore Association for Assessment and Accreditation of Laboratory Animal Care (AAALAC)-accredited facility. All experiments were conducted in compliance with Public Health Service (PHS) guidelines for animal research and approved by UMB Institutional Animal Care and Use Committee. NRG (NOD.Cg-*Rag^tm1Mom^IL2rg^tm1Wjl^/SzJ*) mice were purchased from Jackson Labs (Bar Harbor, Maine) and bred by University of Maryland Veterinary Resources.

To test the MTD of the combination of Ven and PegC, female 6–8 weeks NRG mice (3 mice/group) were dosed with 100 mg/kg Ven (purchased from LC Labs and formulated in 10% DMSO, 30% PEG400, 60% Phosal PG50) orally via gavage 5 days per week, and 250 IU/kg PegC (kind gift of Jazz Pharmaceuticals, formulated in PBS) and their combination. Mice were monitored and weighed 5 days per week.

To test the effect of Ven-PegC on laboratory parameters, CD1 mice were purchased from Envigo (Frederick, MD). Female and male mice 6–8 weeks were dosed either with vehicle (Control) or Ven (75 mg/kg, PO 5 days per week) and/or PegC (200 IU/kg, IV weekly). Mice were euthanized 15 days later and exsanguinated 1 h after last doses. Whole blood and plasma was sent to VRL laboratory (Gaithersburg, MD) for complete blood count (CBC) and clinical chemistries (CMP) including transaminases, bilirubin, pancreatic enzymes and coagulation markers.

A similar experiment was conducted with combination of Ven and pegaspargase (Servier, Boston MA).

### Establishment of the Patient derived xenograft AML45-luc-YFP model

Primary patient cells (AML45) were a kind gift of Drs. Martin Carroll and Alexander Perl (University of Pennsylvania) under an Institutional Review Board-approved research protocol. AML45 cells were derived from a patient with a history of MDS who progressed to AML and later relapsed with a complex karyotype, 46,X,del (X)(p11.4),t(1;3)(p36.1;p25),t(2;?13)(q21;q13),t(2;11) (q12;q25),add(3)(p22),der(3)t(3;?;8)(p22;?;q12.1),der(5) del(5)(p14)t(2;?;5)(q11.2;?;q35),?t(8;16)(p11.2;p13.3), der(15)t(3;?;8)(q24;?;q12.1),t(17;22)(q21;q13). AML45 was transduced with lentivirus to express luciferase and yellow fluorescent protein (YFP). AML45 was seeded at 1 × 10^6^ cells in 500 μL RPMI plus 10% FBS supplemented with 500 nM StemRegenin 1 (SR1, StemCell Technologies, Vancouver, Canada) and 8 mg/mL of polybrene in a 24 well plate. Murine Stem Cell Virus (MSCV)-derived promoter driving luc2 internal ribosome entry site (IRES) YFP lentivirus (kind gift of Dr. Sharyn Baker of The Ohio State University and Viral Vector Core of St. Jude University) was added to the well at a multiplicity of infection (MOI) of 25-30. After 48 h, cells were injected IV into female 6–8 weeks NSG/NRG mice via lateral tail vein. Bone marrows and spleens were collected from mice after 42 days and YFP^+^/huCD33^+^ (Cat. # 551378, BD, Franklin Lakes, NJ) cells were sorted and collected using Fluorescence-activated cell sorting (FACS) on an Aria II (BD Biosciences, San Jose, CA). The transduction / transplantation technique was repeated 3 times until the huCD33^+^ AML45 cells had a YFP^+^ mean fluorescent intensity >10^6^.

Transduced cells were injected IV into female 6–8 weeks NSG (NOD.Cg-*Prkdc^scid^IL2rg^tm1Wjil^/SzJ*) or NRG mice. Mice were imaged on the Xenogen IVIS Spectrum at the Imaging Core of the University of Maryland School of Medicine and after ~100 days, luminescent AML cells were detected. For imaging, mice were injected with 150 mg/kg luciferin (Perkin Elmer, Hopkinton, MA) via intraperitoneal (IP) injection. After ~4 months, mice were euthanized, bone marrow was extracted, and cells were aseptically flushed from femurs and tibias. Bone marrow aspirates were filtered through a 100 μm filter, filtrate was centrifuged at 300 g for 10 min, counted by Trypan blue and reinjected into recipient mice. At the penultimate passage, bone marrow cells were extracted and then aseptically sorted for YFP on the BD FACS Aria II platform in UMGCCC’s flow cytometry core. A pure population of AML45-luc-YFP cells was viably frozen and injected into recipient mice.

### In vivo efficacy in AML45-luc-YFP

Viably frozen AML-45-YFP-luc cells were thawed, washed with PBS and injected into 3 female 6–8 weeks NRG mice and allowed to expand in vivo. After 4 weeks, mice were euthanized, cells isolated from bone marrow aseptically and 1 × 10^6^ AML45-luc-YFP cells were injected IV into NRG mice for the experiment. Three to 10 days later, mice were imaged on the Xenogen IVIS-200 System (Alameda, CA) and robust AML engraftment was confirmed. Mice were sorted into four groups of five with equal mean leukemia burden as measured by luminescence (photon intensity) among the groups. Dosing began on the day of sorting with vehicle, Ven (PO, 75 mg/kg, 5 days per week), PegC (IV, 200 IU/kg, once weekly), or Ven-PegC. Mice were imaged weekly and survival was monitored. Ven was dosed on days 1–5, 8–12, 23–24, 30–33, 78–82, 86–89, and 99–103. PegC was dosed weekly on days 1, 8, 23, 30, 78, 87, and 99. No treatment was administrated between Days 33 and 78. One mouse in the Ven-PegC group died in the second week unrelated to disease or toxicity. Leukemia burden was measured by photon intensity.

Mice were imaged weekly to monitor photon intensity as a surrogate for leukemia burden. Mean photon intensity was calculated by averaging the maximal photon intensity for each mouse on each day of imaging.

### In vivo efficacy in U937-luc

U937 cells were cultured in RPMI 1640 supplemented with 10% FBS and 1X Glutamax, per ATCC instructions. On the day of transduction, actively growing cells were counted and seeded into a 24-well plate at 105 cells per 500 μL. Cells were transduced with YFP-Luciferase lentiviral supernatant for 72 h in the presence of 8 μg/mL polybrene. Cells were then harvested, washed once with PBS, and plated into T25 cell culture flasks for propagation. Actively growing, transduced cells were subjected to sterile sorting using an Aria II. U937-luc cells (0.25 × 10^6^) were injected IV into female 6–8 weeks NRG mice. Three days post cell injection, mice were imaged and engraftment was confirmed. Mice were sorted into 6 groups of 5 mice (control, Ven, PegC, Ven-PegC, Aza, and Aza-Ven) with equal mean leukemia burden among the groups. Treatment started on the day of sorting. Following engraftment, treatment was initiated with Ven (75 mg/kg PO 5 days weekly) and/or PegC (200 IU/kg IV weekly) and/or azacitidine (0.5 mg/kg subcutaneous 5 days weekly). Ven was dosed on days 1–5, 8–10. PegC was dosed on days 1, 8. Aza was dosed on days 2–5, 8–12. In the Ven-PegC group (the only mice to live beyond day 18), mice were treated with one more dose of PegC and 3 more doses of Ven. Mice were imaged weekly and survival was monitored. Leukemia burden was measured by photon intensity.

### Pharmacodynamic assay

AML45-luc cells (0.5 × 10^6^) were injected IV into female 6–8 weeks NRG mice. Mice were dosed with vehicle, Ven days 1–5, 8–12, 15–19, 29–33, and 36–38), PegC (days 1, 8, 15, 29, and 36) and Ven-PegC at doses similar to the efficacy study. On day 39 post start of dosing, mice were euthanized. Plasma was isolated from whole blood after exsanguination and delivered on ice to the Biochemical Genetics lab (University of Maryland Baltimore) to measure plasma glutamate, glutamine, and asparagine. Bone marrow cells were extracted from both femurs as described above. Cells were pelleted and lysed in RIPA buffer (above) and used for western blot analysis.

Free amino acid (AA) concentrations were measured via a dedicated high-pressure liquid chromatography (HPLC) AAanalyzer (Biochrom 20 PlusAmino or Biochrom 30 Acid Analyzer). Free AAs were separated with a cation-exchange resin post precipitation of protein with sulfosalicylic acid. The AAs were differentially eluted after increasing the pH and ionic strength of the buffer. The concentration of each AA was measured by reaction of the AA with ninhydrin and detection via a colorimeter. Standard curves were generated for each AA and AAs were quantified. The results were reported as μmol/L (μM) of AA in plasma. Two quality control standards were evaluated as unknowns at the beginning of each set of sample runs. To minimize continued hydrolysis of glutamine and asparagine by PegC ex vivo before their measurement by HPLC, blood obtained in a sodium or lithium heparin tube was wrapped in a paper towel and placed in a plastic biohazard bag, which was set on ice without placing the tube directly in ice to avoid hemolysis. The bag was transferred to the laboratory in less than 20 min, the plasma was separated (spun at 1100 g × 5 min at 4 °C), aliquoted, and frozen at −20°C until analysis.

### Statistical analysis

For IC_50_s in the AML cell lines, data are presented as means ± standard deviations (SD) and *p* < 0.05 was considered as significant. For the in vitro mechanistic studies, data are presented as means ± standard error of means (SEM) with *p* < 0.05 as significant.

To compare the growth rate of engrafted tumors by study arm, we drew line graphs of photon intensity vs. follow-up time for each mouse and compared the study arms using linear random effects models. Similarly, weights were charted for each of the mice, as a percentage of their initial weight, vs. follow-up time.

Survival of mice with engrafted AML cells was estimated using Kaplan–Meier estimators and compared across the study arms using log-rank tests. The outcome of interest for this survival analysis was death due to leukemia, i.e., only if photon intensity reached the critical level of 1.5 × 10^8^. Death due to reasons other than leukemia (indicated by photon intensity <1.5 × 10^8^) were censored at the date of death.

For mechanistic studies that needed comparisons of means across multiple treatment groups (e.g., to compare alteration of gene transcription in AML after treatment with Ven, PegC, and Ven-PegC), we used analysis of variance (ANOVA) followed by Bonferroni’s post hoc corrections. Statistical parameters for each experiment including sample size and statistical significance are reported in the figures and corresponding figure legends. In vivo sample size was determined based on achieving 80% power with a type I error rate of 5% and an anticipated difference of ~20% in mean leukemia burden between arms.

To compare plasma concentrations of glutamine, asparagine, and glutamate between the two groups of mice that did or did not receive PegC, we used Mann–Whitney *U* tests.

## Supplementary Material

Supplemental material

Table S5
