# Supplementary material for: Venetoclax and pegcrisantaspase for complex karyotype acute myeloid leukemia
Source: Leukemia. Author manuscript; Available in PMC 2024 Mar 28. (PMC10976320; doi:10.1038/s41375-020-01080-6)
Supplement: Supplemental material [file NIHMS1973698-supplement-Supplemental_material.docx]

**Supplementary Materials and Methods**

***Supplementary Figures***

Supplementary Fig. S1. Representative growth inhibition curves induced by PegC for each CK-AML cell line.

Supplementary Fig. S2. Effect of Ven, PegC and their combination on cell cycle.

Supplementary Fig. S3. Effect of Ven and/or PegC on anti-apoptotic proteins.

Supplementary Fig. S4. Neither Ven nor PegC potentiates anti-AML activity of decitabine or azacitidine in two human AML cell lines.

Supplementary Fig. S5. Ven-PegC was well tolerated in NRG mice.

Supplementary Fig. S6. Photon intensity (leukemia burden) and weight changes in CK-AML *in vivo* models.

Supplementary Fig. S7. Alignment statistics and heatmap for transcriptome samples.

Supplementary Fig. S8. Correlation of gene regulation when exposed to Ven, PegC and Ven-PegC.

Supplementary Fig. S9. qRT-PCR analysis of expression of indicated genes in MOLM-14 cells treated with PegC, Ven and Ven-PegC combination.

Supplementary Fig. S10. Ven-PegC inhibits proteins required cap-dependent translation in MonoMac6 cells.

Fig. S11. qRT-PCR analysis of expression of the indicated genes in treated bone marrow cells.

***Supplementary Tables***

Supplementary Table S1. Cytogenetics and Mutational Characteristics of Human AML Cell Lines with Complex Karyotype.

Supplementary Table S2. Effect of Ven + Pegaspargase on blood counts and organ function of interest

Supplementary Table S3. Effect of Ven + Pegaspargase on blood counts and organ function of interest.

Supplementary Table S4. Transcriptome alignment summary.

Supplementary Table S5. Transcriptome data analysis [Separate Excel file]

Supplementary Table S6. KEGG and GO analysis.

Supplementary Table S7. Twenty three genes modulated by Ven and PegC and Ven-PegC.

Supplementary Table S8. Key Resources.


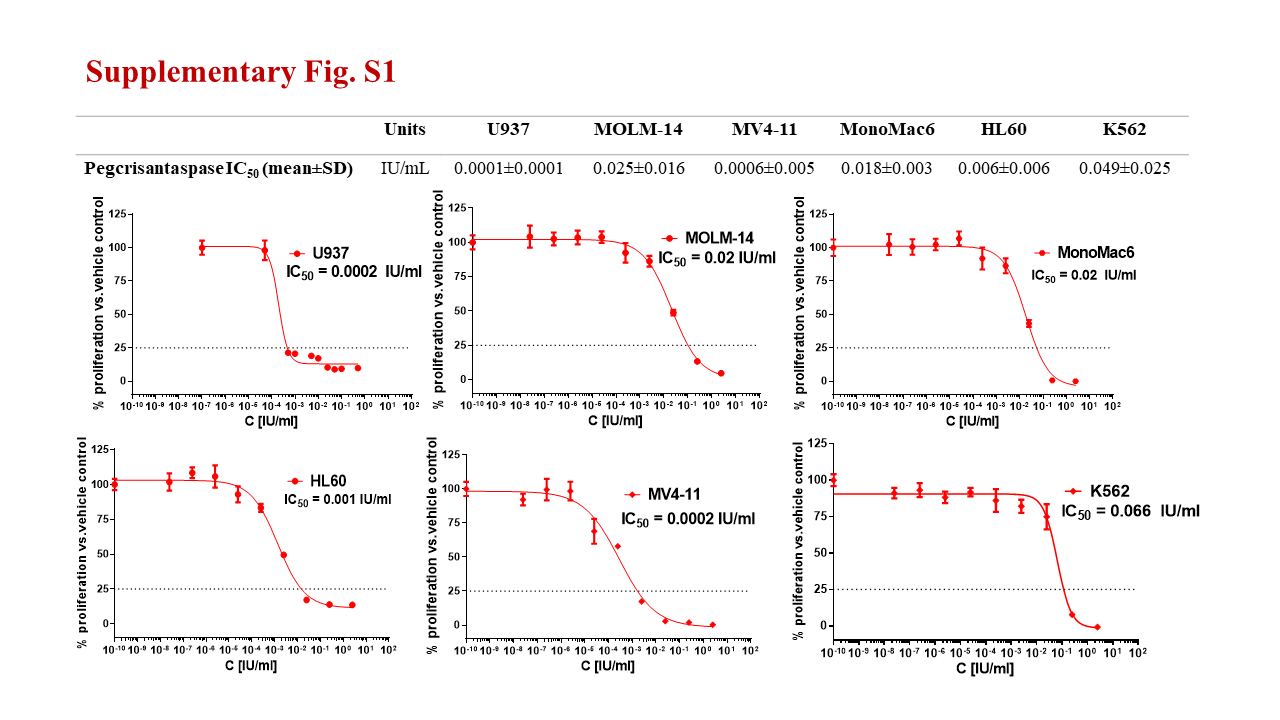


**Supplementary Fig. S1. Representative growth inhibition curves induced by PegC for each CK-AML cell line.** Approximately 18h after plating, human AML cell lines were treated for 72h with PegC. Proliferation of cells was measured by addition of mitochondrial dye, WST-1 (water-soluble tetrazolium salts, Takara Bio USA, Inc., TBUSA, formerly known as Clontech Laboratories, Inc., Mountain View, CA). IC_50_s were generated by GraphPad Prism. IC_50_s were estimated from 3-4 repeat experiments for each AML cell line. SD = standard deviation

**
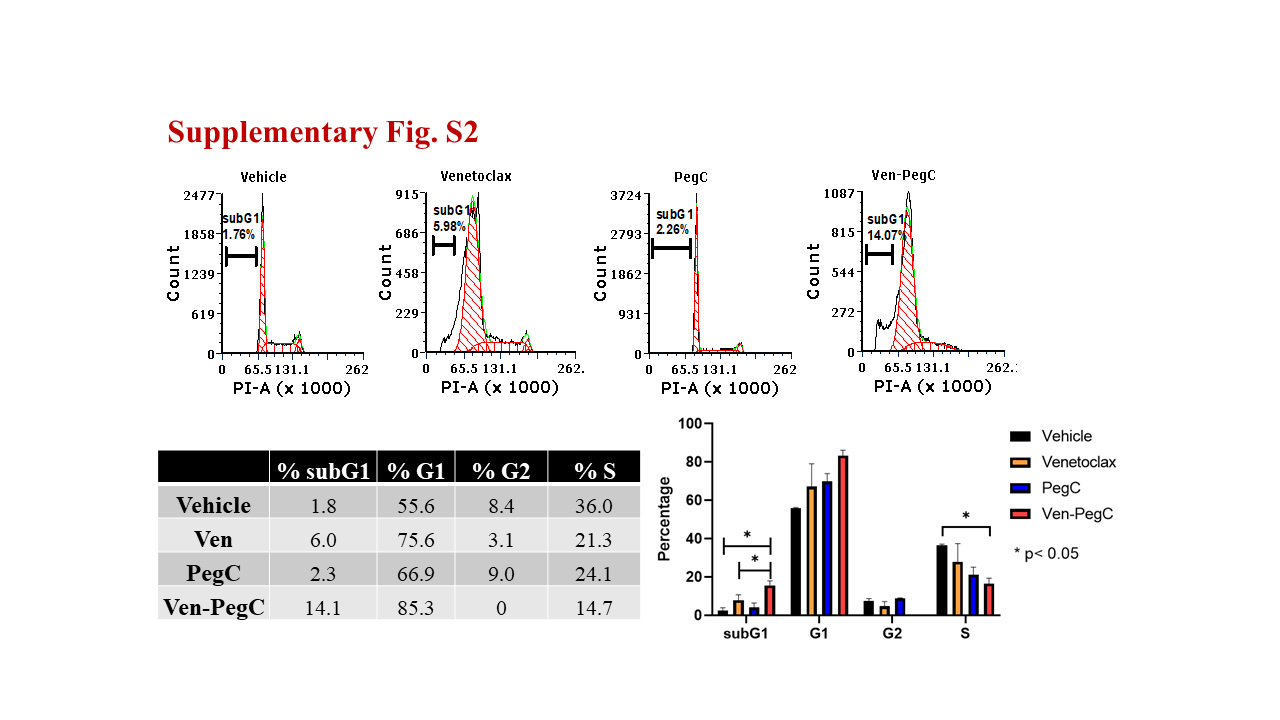
**

**Supplementary Fig. S2. Effect of Ven, PegC and their combination on cell cycle.** AML cells (MOLM-14) treated with vehicle (DMSO), Ven, PegC and Ven-PegC at IC_50_ for 48h. Cells were fixed in ethanol, stained with propidium iodide (PI), then analyzed by flow cytometry. Representative histograms are shown (top) along with the corresponding percentage of cells in each cell cycle phase (bottom left) and the results from two independent experiments are shown in the bottom right panel.

**
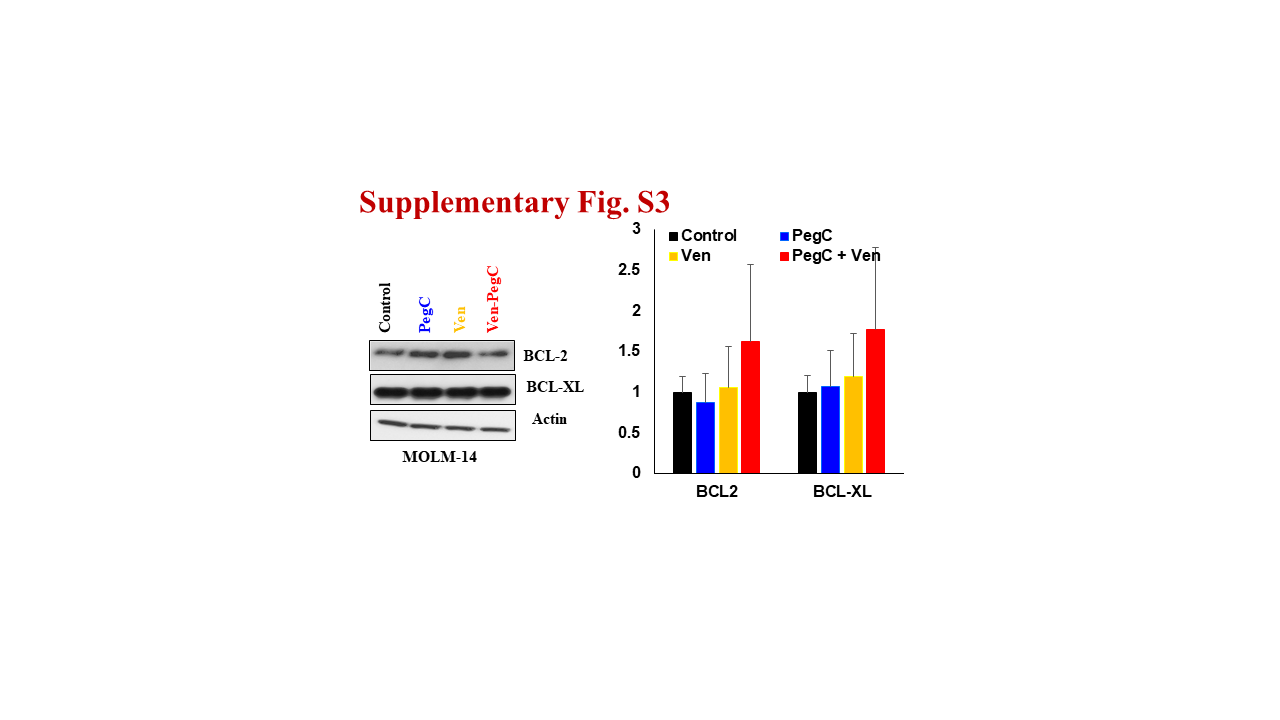
**

**Supplementary Fig. S3. Effect of Ven and/or PegC on anti-apoptotic proteins.** MOLM-14 cells were treated with Ven, PegC or Ven-PegC at corresponding IC_50_s for 24h. Cell lysates were prepared, followed by western blot analysis for the anti-apoptotic proteins BCL-2 and BCL-XL.


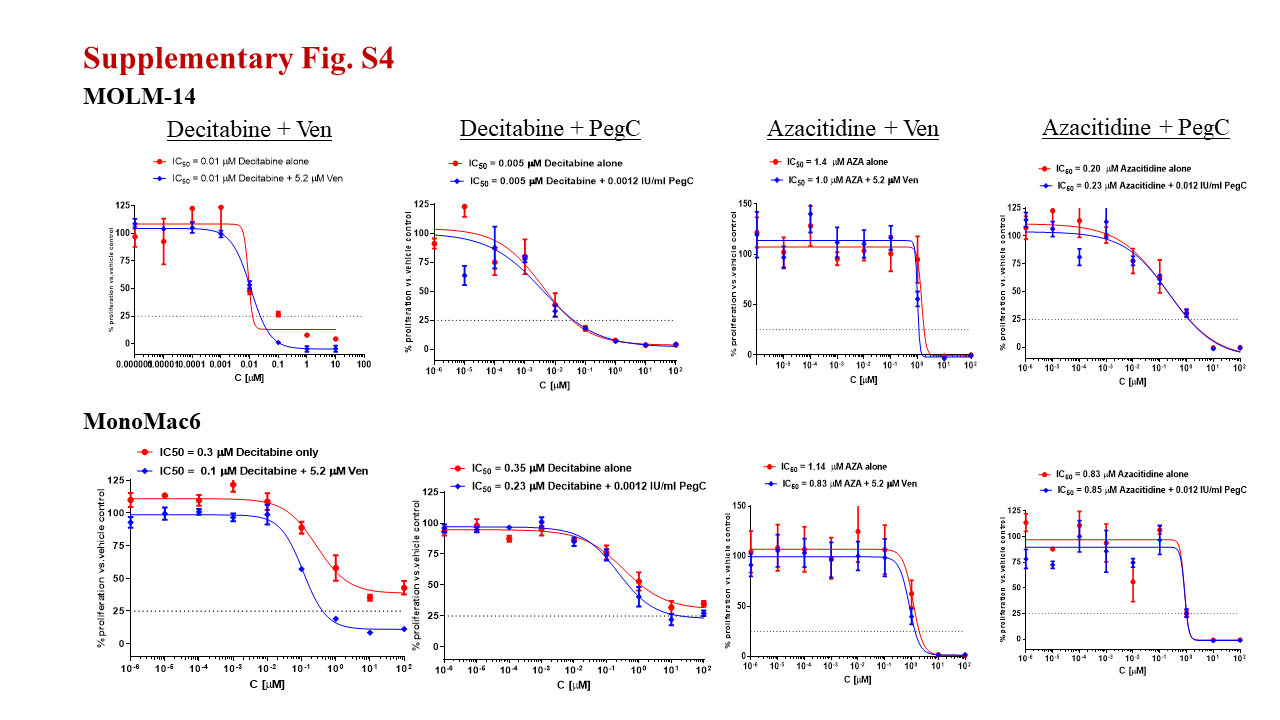


**Supplementary Fig. S4.** **Neither Ven nor PegC potentiates anti-AML activity of decitabine or azacitidine in two human AML cell lines.** MOLM-14 and MonoMac6 cells were exposed to decitabine or azacitidine at a range of concentrations in the presence or absence of Ven or PegC at the corresponding single-agent IC_50_s for 72h. IC_50_ values were generated by GraphPad Prism.


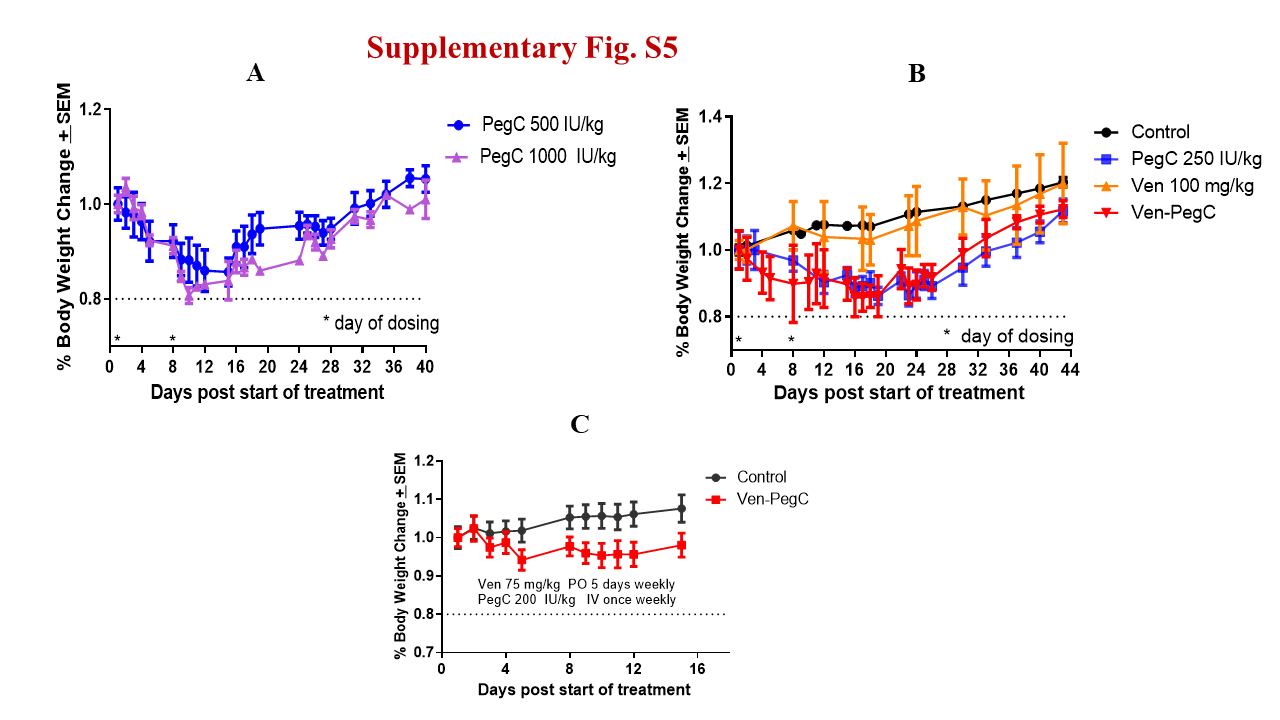


**Supplementary Fig. S5.** **Ven-PegC was well tolerated in NRG mice.** **(A)** NRG mice were dosed with 1000 IU/kg or 500 IU/kg PegC IV once per week for two weeks. **(B)** NRG mice were dosed with 250 IU/kg PegC IV weekly for two weeks alone or in combination with 100 mg/kg Ven dosed PO 5 days per week for two weeks. In both studies, mice were observed 5 days per week and monitored for an additional four weeks post dosing. **(C)** The MTD of Ven-PegC combination was determined by showing no significant weight loss.


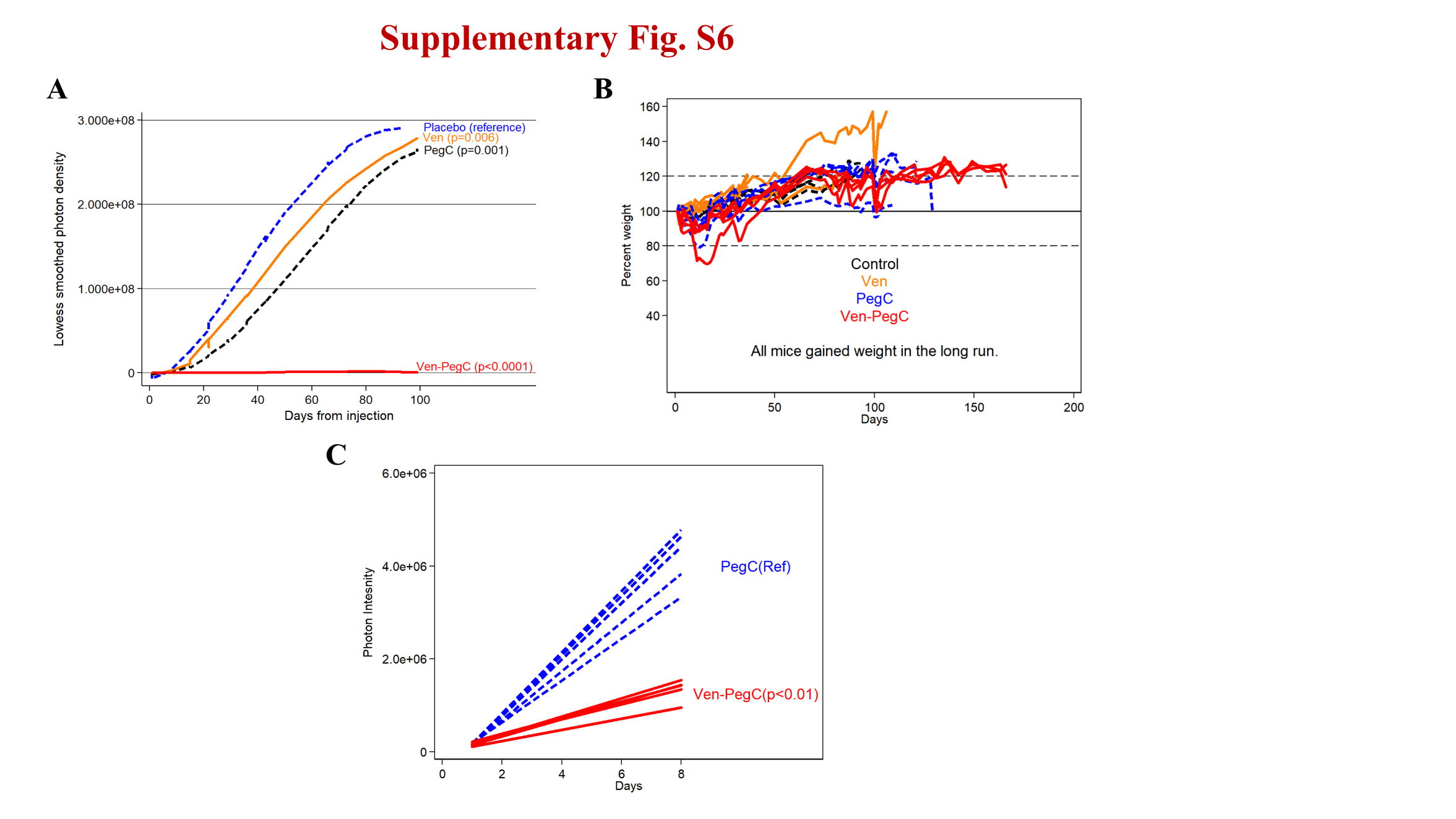


**Supplementary Fig. S6. Photon intensity (leukemia burden) and weight changes in CK-AML *in vivo* models. (A)** Lowess (locally weighted scatter plot smoother) graph of photon intensity an orthotopic patient-derived xenograft (PDX) model of relapsed AML with complex karyotype (AML45-luc), versus time after administration of Vehicle, Ven, PegC and Ven-PegC combination. In mice treated with Ven-PegC combination, photon intensity remains virtually constant over 100 days. **(B)** Percent weight changes versus time. Mice recovered after initial weight loss, and all gained weight in long term. **(C)** Photon intensity in U937-luc versus time. Compared to PegC, photon intensity in Ven-PegC is statistically significantly less. Each line represents a different mouse.

**
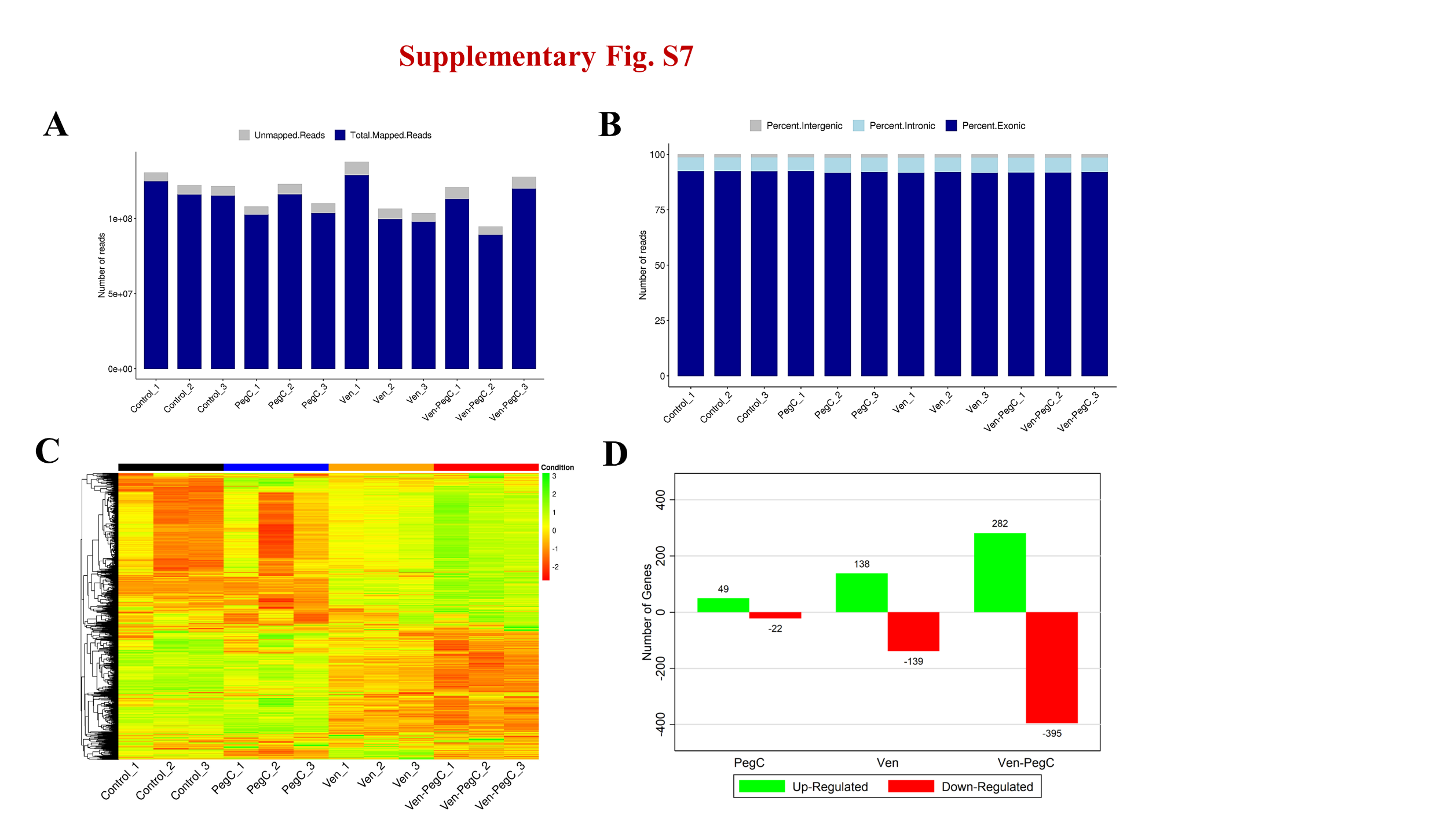
**

**Supplementary Fig. S7. Alignment statistics and heatmap for transcriptome samples.** The bar plot summarizes the alignment statistics for the transcriptome dataset. **(A)** The number of reads that mapped (blue) and did not map (grey) to the human reference genome for each sample. On average, ~95% of the reads mapped to the human reference genome. **(B)** The proportion of mapped reads that aligned to different regions in the genome, including exonic (dark blue), intronic (light blue), and intergenic (grey) regions. On average, ~92% of the mapped reads aligned within exonic regions across all of the transcriptome samples, consistent with a high-quality transcriptome. **(C)** Heatmap of differentially expressed genes for MOLM14 transcripts treated with Ven (5.2 µM) and/or PegC (0.025 IU/mL) for 16h. DMSO was used as control. Unregulated genes are shown, ranked by log2 fold-change (FC), log2 FC < -1 or log 2 FC > 1, and adjusted p<0.05 relative to control. **(D)** Bar plots showing the number of genes significantly altered (green upregulated, red downregulated) compared with cells treated with vehicle control. Protein coding transcripts were a predominant feature detected in all treatment conditions.

**
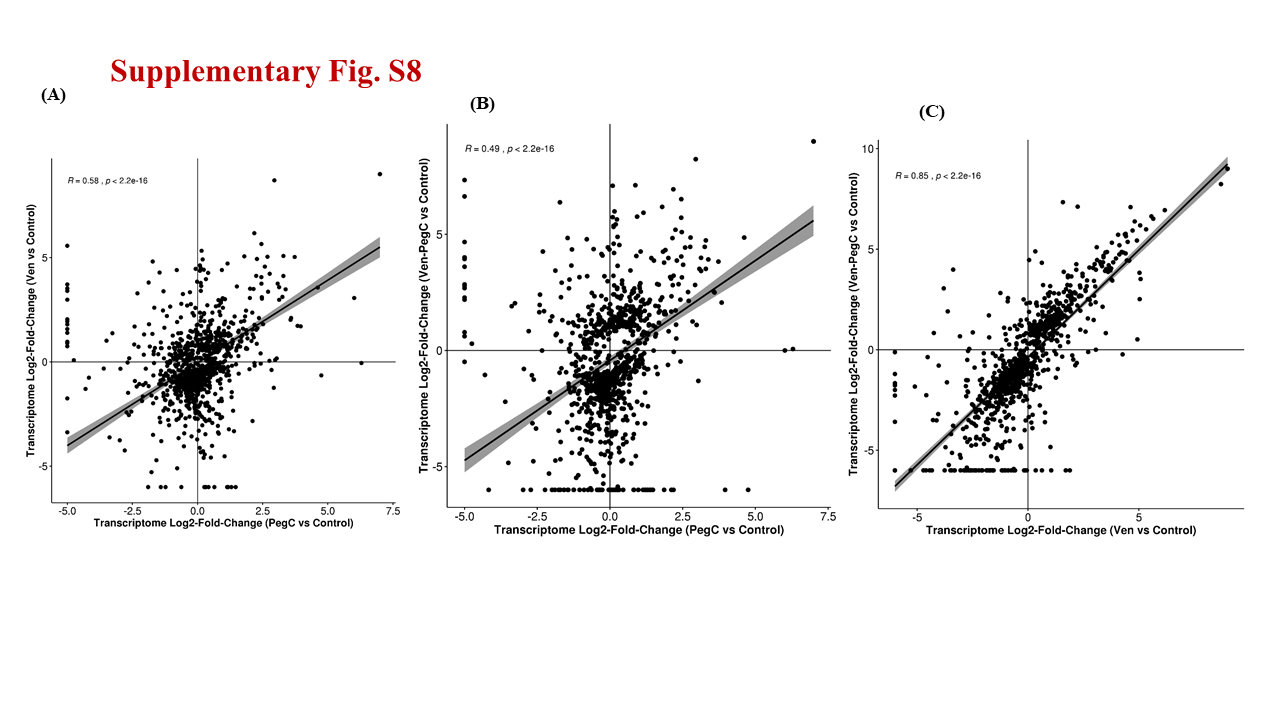
**

**Supplementary Fig. S8. Correlation of gene regulation when exposed to Ven, PegC and Ven-PegC.** The scatterplots illustrate the correlation between the differential gene expression observed with treatment with one drug (PegC or Ven) compared to treatment with both drugs (Ven-PegC). Each point represents the log2 (fold-change) estimate for a gene from a single drug treatment or the combination treatment when compared to the control (DMSO). The correlation estimates and p-values are computed using the Pearson’s correlation method implemented in R statistical package.

**
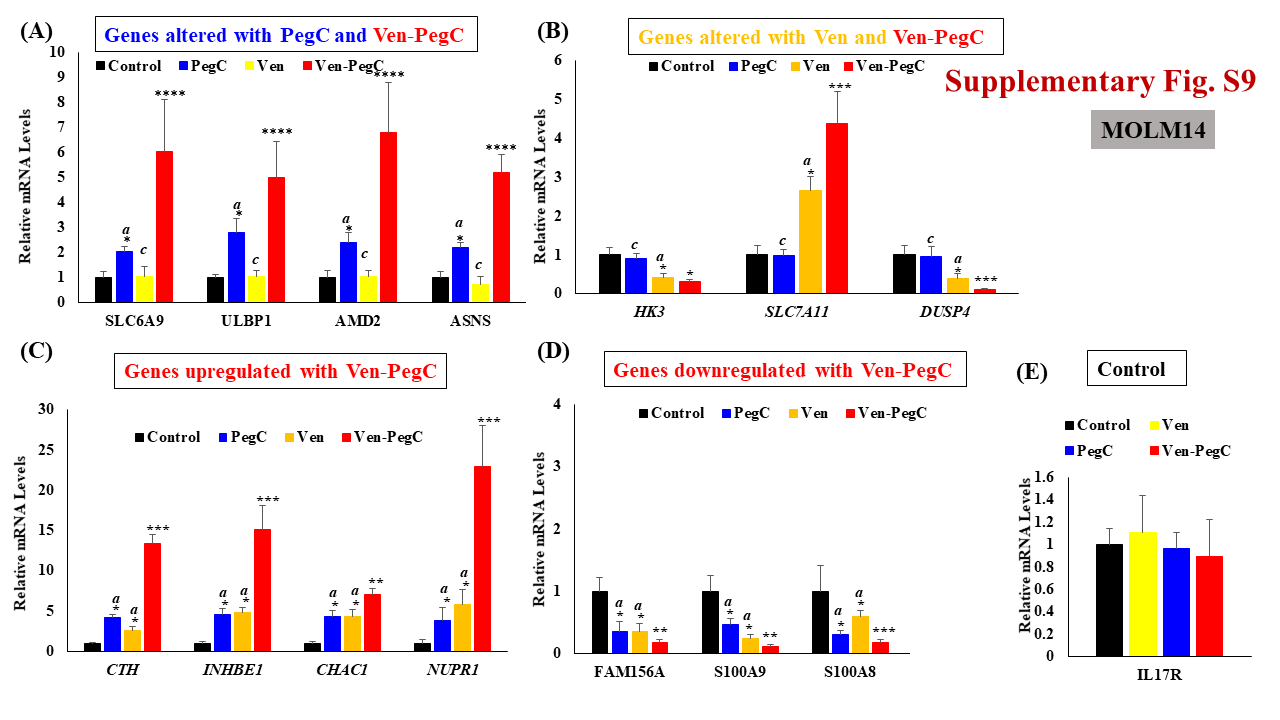
**

**Fig. S9. qRT-PCR analysis of expression of indicated genes in MOLM-14 cells treated with PegC, Ven and Ven-PegC combination (A-D).** qRT-PCR of selected genes modified by Ven-PegC, compared with the control gene IL17R **(E)**, confirmed transcriptome analysis results. Results were normalized to corresponding control-treated cells and expressed as mean±SD (n=3). Statistical analysis was performed using one-way ANOVA, and p-values were adjusted using Bonferroni's correction method *p<0.05, **p<0.01, ***p<0.005 vs corresponding control-treated cells, ^a^p<0.05, ^b^p<0.01, ^c^p<0.005 vs corresponding Ven-PegC-treated cells.


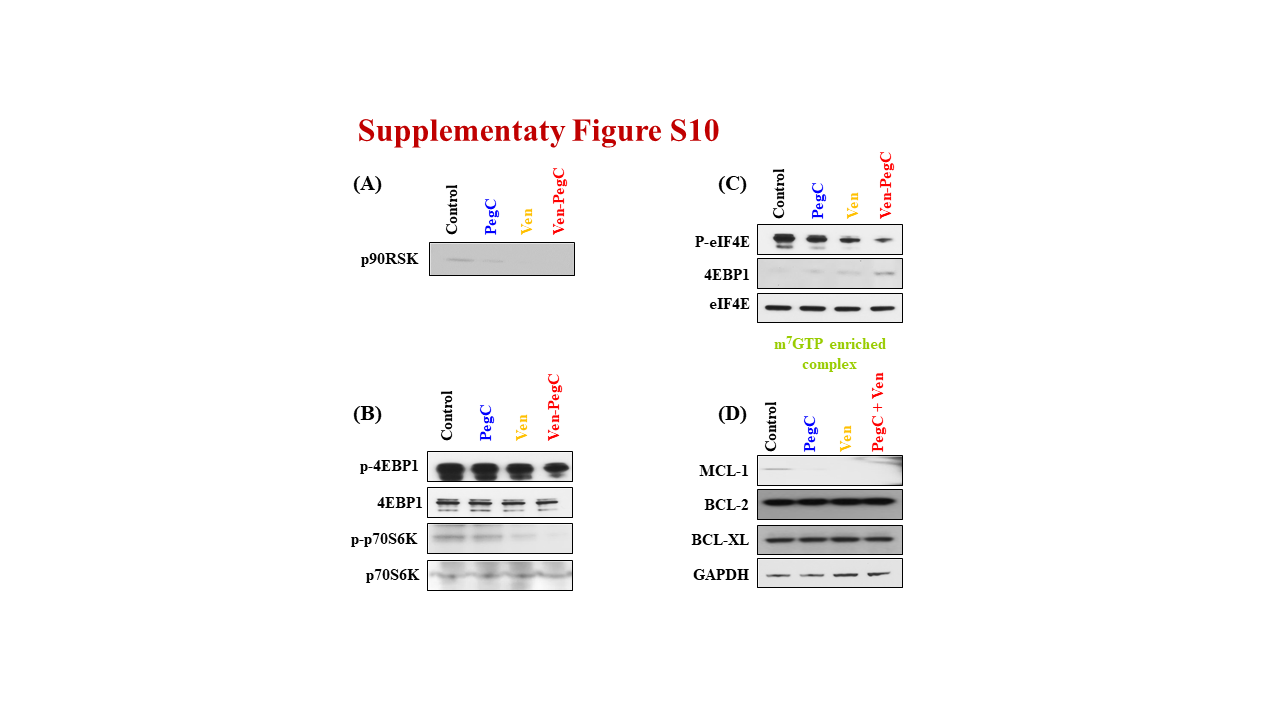


**Supplementary Fig. S10. Ven-PegC inhibits proteins required cap-dependent translation in MonoMac6 cells.** (A, B and D) MonoMac6 were treated similarly to MOLM-14 cells with PegC, Ven and Ven-PegC (see Fig. 5). Cellular lysates were probed with the indicated antibodies. GAPDH loading control in D applies to panels A and C as well. (C) Precleared cellular lysates of MonoMac6 cells treated with PegC, Ven and Ven-PegC were incubated with m^7^GTP sepharose beads for 2h followed by washing and probing with the indicated antibodies.


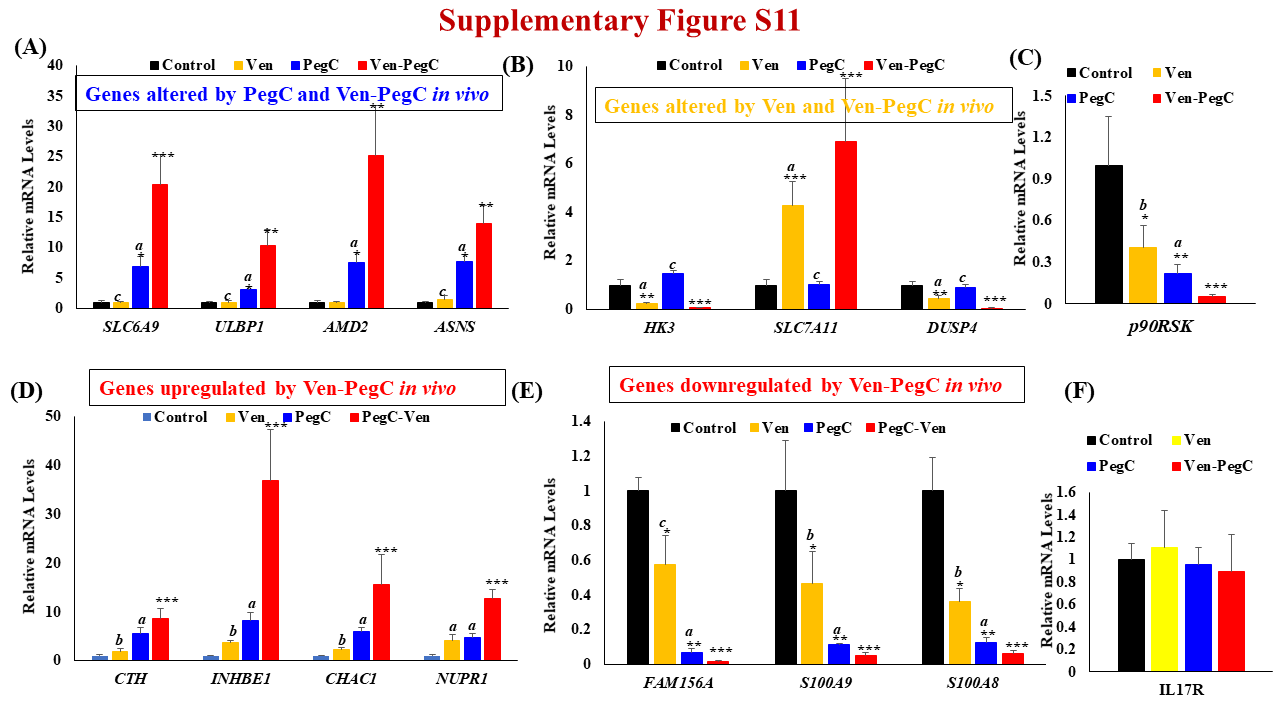


**Supplementary Fig. S11. qRT-PCR analysis of expression of the indicated genes in treated bone marrow cells.** Bone marrow cells of mice treated with PegC, Ven and Ven-PegC combination. These *in vivo* results were similar to the *in vitro* results in Supplementary Fig. S9. Results were normalized to corresponding control-treated cells and expressed as mean±SD (n=3). Statistical analysis was performed using one-way ANOVA, and p-values were adjusted using Bonferroni's correction method *p<0.05, **p<0.01, ***p<0.005vs corresponding control-treated cells, ^a^p<0.05, ^b^p<0.01, ^c^p<0.005vs PegC-Ven treated corresponding cells.

| **Table S1. Cytogenetics and Mutational Characteristics of Human AML Cell Lines** | | | | |
| --- | --- | --- | --- | --- |
| **Cell Line** | **Karyotype** | **Molecular analyses** | | |
|  |  | **STR** | **FLT3** | **Other Mutations** |
| **U937** | 56-60,X,+X,-Y,der(1)t(1;5)(p22;q31),+2,der(2)dup(2)(q31q33)t(2;6)(q33; q21),+3,der(3)(1;3)(q12;q26),der(5)t(1;5)(p22;q31),+6,+6,der(6)t(2;6)(p13; p21),der(6)del(6)(p22)dup(6)(p12p22),+7,+8,der(10)dup(q24q25)t(10;11) (p13;q14),der(11)t(10;11)(p13;q14),+12,dic(6;12)(q10;p10),der(13)t(1;13) (q10;q10),ider(13)(q14),+15,+15,der(15)inv(9)(q15q25)del(15)(q11q15) del(15)(q25),der(16)t(4;16)(p13;p13),+18,+20,+21 | Matched to database | FLT3-ITD -, FLT3 TKD - | DNMT3A Q249fs, GATA2 P161A, PTPN11 G60R, WT1 R369* |
| **MOLM-14** | 50-51,XY,t(2;5)(p23;q11.2),+5,der(5;19)(p10;q10),+6,der(6)t(1;6)(q21; q27), +8,i(10)(q10), ins(11;9)(q23.3;p22p23),+12,i(12)(p10),i(12)(q10),+13, del(16)(q11.2q13.1),+19,del(19)(q13.1q13.3) | Matched to database | FLT3-ITD +, FLT3 TKD - | ASXL1 G652S, FLT3 T227M, KMT2A A1653T, SETBP1 V1101I, SETBP1 P1130T, TET2 I1762V, TET2 V218M |
| **MV4-11** | 47-49,XY, t(4;11)(q21;q23),+8,del(9)(q21q32),add(10)(p13),+13,+19 | Matched to database | FLT3-ITD +, FLT3 TKD - | FLT3 D7G, SETBP1 V1101I, TET2 I1762V, TP53 P219fs |
| **MonoMac-6** | 81-84<4n>,XX/XXX,-Y,i(1)(q10),add(7)(p21),t(9;11)(p21;q23)x2,-12,-13,-13,der(13;14)(q10;q10)x2,-16,-16, -17, -17, +3-6mar | Matched to database | FLT3-ITD -, FLT3 TKD - | DNMT3A T251fs, DNMT3A V258fs, FLT3 T227M, KMT2A P2357R, NRAS G12D, SETBP1 V1101I, TP53 R174fs |
| **HL-60** | 45-47,X,-X,add(1)(p13),-2,del(3)(p21.3),add(3)(q29),add(4)(q35), add(5)(q33),del(9)(q32),add(11)(p13),add(13)(q34),-14,add(15)(p11.2),-16, add(16)(q22),-17,+4-6mar | Matched to database | FLT3-ITD -, FLT3 TKD - | CSF3R E149D, FLT3 T227M, GATA2 A164T, IDH1 V178I, NRAS Q61L |
| **K562** | 62-69<3n>,XX,-X,-3,del(3)(q12),add(6)(p23),+7,add(7)(p15),-9,del(9)(p13), add(9)(p24),der(10)t(3;10)(p21.3;q23)x1-2,-13,-14,add(17)(p11.2)x2, der(18)t(1;18)(p32;q21),der(20)t(1;6;20)(?;?;p11.1),-21,der(21)t(1;21) (q23;p11),+2-4mar | Matched to database | FLT3-ITD -, FLT3 TKD - | ASXL1 Y591*, CBL D792H, FLT3 T227M, NOTCH1 G326V, SETBP1 P1130T, SETBP1 V231L, TET2 L1721W, TET2 I1762V, TP53 Q136fs |
|  |  |  |  |  |
|  |  |  |  |  |


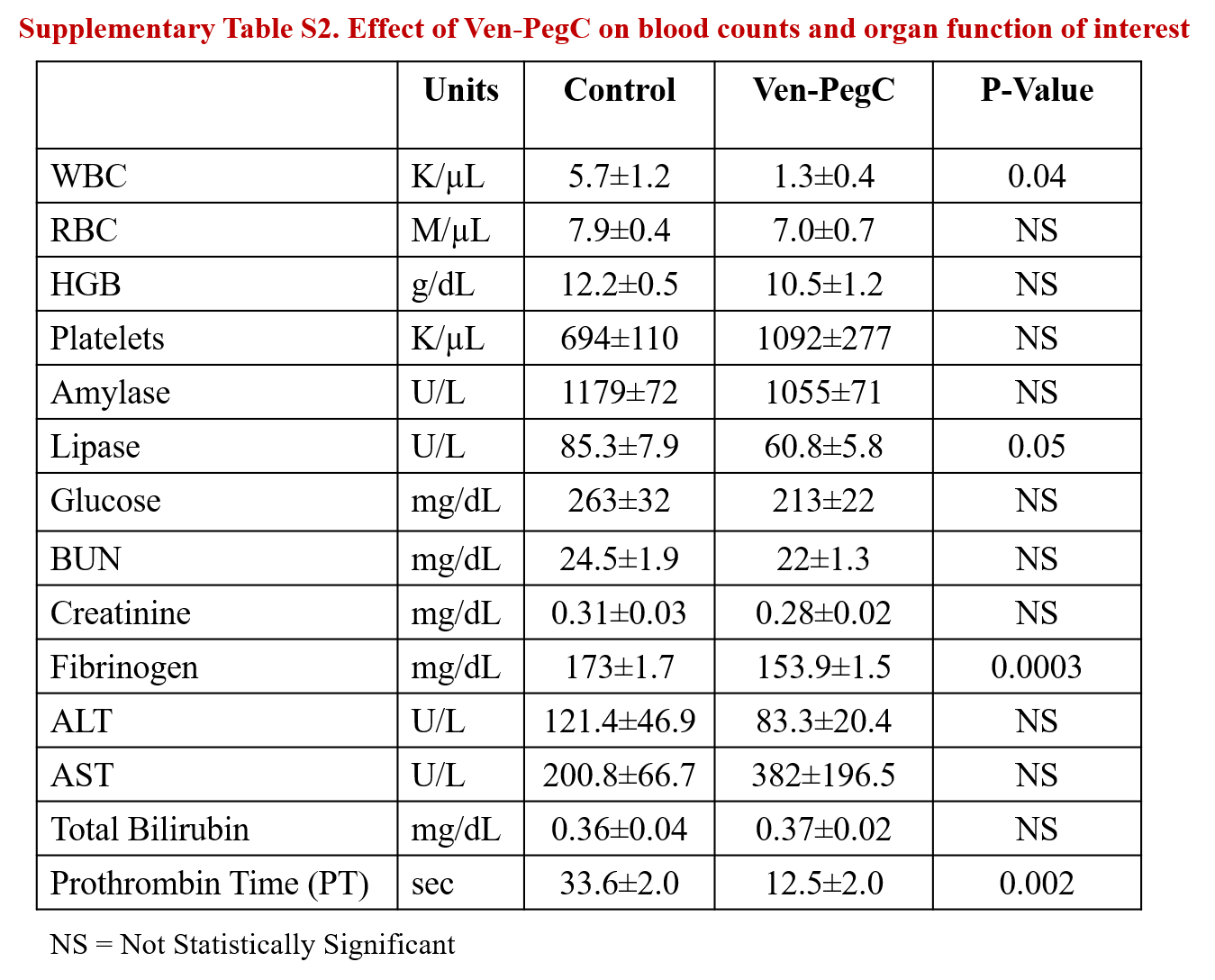

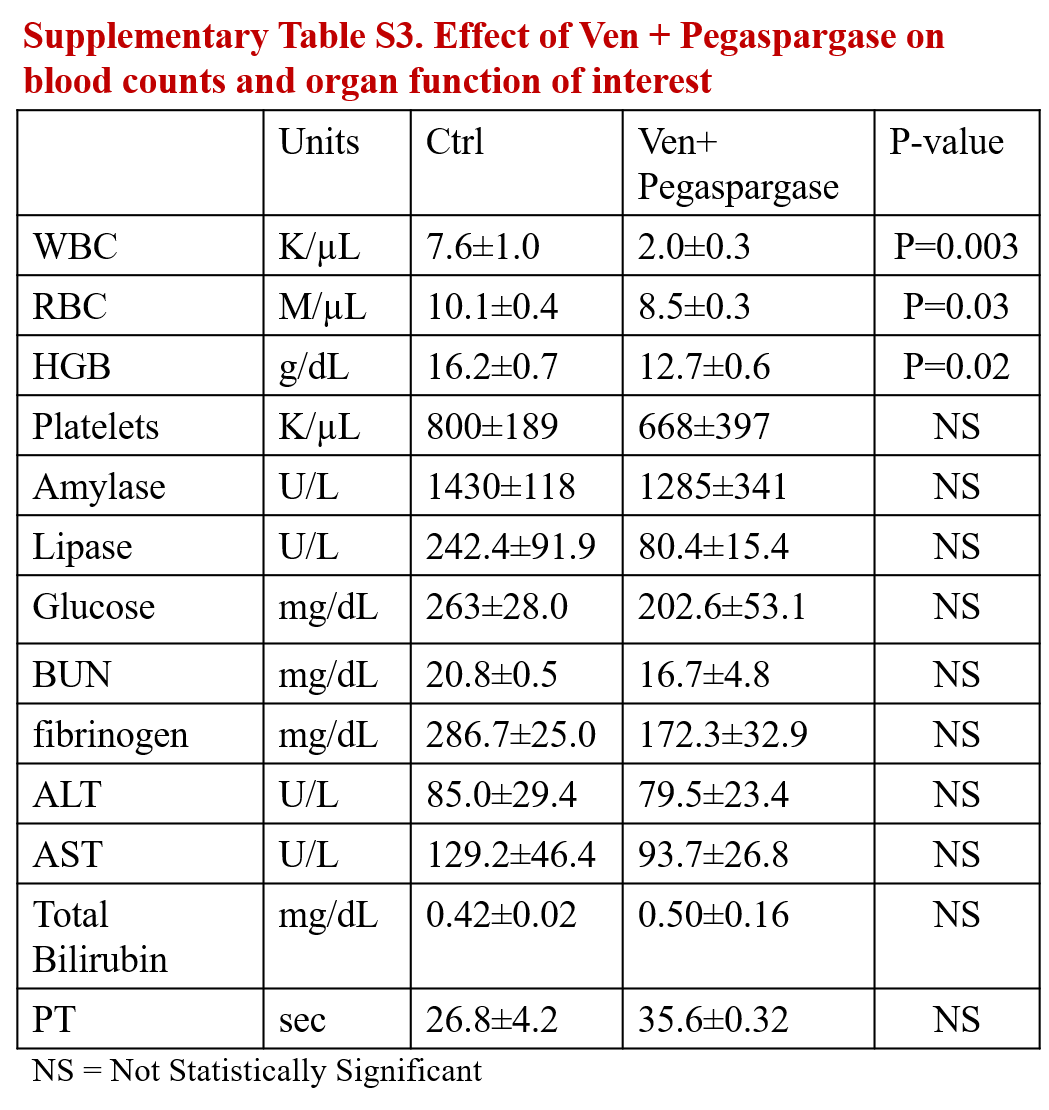


| **Supplementary Table S4. Transcriptome alignment summary** | | | | | | | | | | | |
| --- | --- | --- | --- | --- | --- | --- | --- | --- | --- | --- | --- |
| **#Sample_ID** | **Total Reads** | **Total Mapped Reads** | **Percent Mapped Reads** | **Percent Properly Paired** | **Uniquely Mapped Reads** | **Percent Exonic** | **Percent Intronic** | **Percent Intergenic** | **Total Features** | **Features With Coverage** | **Avg RPKM** |
| **Control_1** | 130,570,166 | 125,051,172 | 95.77 | 95.06 | 121,963,833 | 92.67 | 6.19 | 1.14 | 58,302 | 32,104 | 4.501 |
| **Control_2** | 122,116,112 | 116,281,226 | 95.22 | 94.40 | 112,920,868 | 92.67 | 6.17 | 1.17 | 58,302 | 31,309 | 4.667 |
| **Control_3** | 121,534,284 | 115,556,054 | 95.08 | 94.28 | 112,073,463 | 92.60 | 6.24 | 1.16 | 58,302 | 31,672 | 4.602 |
| **PegC_1** | 107,909,424 | 102,840,996 | 95.30 | 94.41 | 99,726,311 | 92.72 | 6.14 | 1.14 | 58,302 | 31,851 | 4.539 |
| **PegC_2** | 106,441,872 | 99,895,962 | 93.85 | 93.21 | 96,410,456 | 92.24 | 6.54 | 1.22 | 58,302 | 31,641 | 4.792 |
| **PegC_3** | 109,946,180 | 103,866,025 | 94.47 | 94.13 | 100,398,530 | 92.24 | 6.53 | 1.23 | 58,302 | 32,638 | 4.553 |
| **Ven_1** | 137,676,798 | 129,196,350 | 93.84 | 93.05 | 125,244,716 | 91.94 | 6.77 | 1.29 | 58,302 | 31,820 | 4.626 |
| **Ven_2** | 122,864,194 | 116,422,827 | 94.76 | 93.80 | 113,626,487 | 91.91 | 6.79 | 1.31 | 58,302 | 31,647 | 4.530 |
| **Ven_3** | 103,433,650 | 98,164,185 | 94.91 | 94.23 | 95,508,101 | 91.87 | 6.87 | 1.25 | 58,302 | 31,362 | 4.566 |
| **Ven-PegC_1** | 120,734,540 | 113,293,891 | 93.84 | 93.69 | 109,966,197 | 92.05 | 6.66 | 1.28 | 58,302 | 30,465 | 4.501 |
| **Ven-PegC_2** | 94,593,376 | 89,438,598 | 94.55 | 94.61 | 86,394,207 | 92.02 | 6.68 | 1.30 | 58,302 | 30,824 | 4.661 |
| **Ven-PegC_3** | 127,679,692 | 120,201,691 | 94.14 | 93.98 | 116,291,116 | 92.24 | 6.52 | 1.24 | 58,302 | 31,115 | 4.620 |

| **Supplementary Table S7. 23 genes modulated by Ven and PegC and Ven-PegC** | | |
| --- | --- | --- |
| **Ensembl ID** | **Gene Name** | **Functional Description** |
| ENSG00000071242 | **RPS6KA2** | ribosomal protein S6 kinase A2 [Source:HGNC Symbol;Acc:HGNC:10431] |
| ENSG00000104921 | **FCER2** | Fc fragment of IgE receptor II [Source:HGNC Symbol;Acc:HGNC:3612] |
| ENSG00000113739 | **STC2** | stanniocalcin 2 [Source:HGNC Symbol;Acc:HGNC:11374] |
| ENSG00000116761 | **CTH** | cystathionine gamma-lyase [Source:HGNC Symbol;Acc:HGNC:2501] |
| ENSG00000124194 | **GDAP1L1** | ganglioside induced differentiation associated protein 1 like 1 [Source:HGNC Symbol;Acc:HGNC:4213] |
| ENSG00000128965 | **CHAC1** | ChaC glutathione specific gamma-glutamylcyclotransferase 1 [Source:HGNC Symbol;Acc:HGNC:28680] |
| ENSG00000139269 | **INHBE** | inhibin beta E subunit [Source:HGNC Symbol;Acc:HGNC:24029] |
| ENSG00000142405 | **NLRP12** | NLR family pyrin domain containing 12 [Source:HGNC Symbol;Acc:HGNC:22938] |
| ENSG00000143546 | **S100A8** | S100 calcium binding protein A8 [Source:HGNC Symbol;Acc:HGNC:10498] |
| ENSG00000163220 | **S100A9** | S100 calcium binding protein A9 [Source:HGNC Symbol;Acc:HGNC:10499] |
| ENSG00000176046 | **NUPR1** | nuclear protein 1, transcriptional regulator [Source:HGNC Symbol;Acc:HGNC:29990] |
| ENSG00000177144 | **NUDT4P1** | nudix hydrolase 4 pseudogene 1 [Source:HGNC Symbol;Acc:HGNC:18012] |
| ENSG00000196517 | **SLC6A9** | solute carrier family 6 member 9 [Source:HGNC Symbol;Acc:HGNC:11056] |
| ENSG00000233024 | **AC126755.2** | nuclear pore complex-interacting protein family member A5 isoform 2 [Source:RefSeq peptide;Acc:NP_001338129] |
| ENSG00000233476 | **EEF1A1P6** | eukaryotic translation elongation factor 1 alpha 1 pseudogene 6 [Source:HGNC Symbol;Acc:HGNC:3201] |
| ENSG00000246100 | **LINC00900** | long intergenic non-protein coding RNA 900 [Source:HGNC Symbol;Acc:HGNC:27444] |
| ENSG00000250535 | **STK19B** | serine/threonine kinase 19B (pseudogene) [Source:HGNC Symbol;Acc:HGNC:21668] |
| ENSG00000251495 | **PPIAP11** | peptidylprolyl isomerase A pseudogene 11 [Source:HGNC Symbol;Acc:HGNC:9263] |
| ENSG00000263740 | **RN7SL4P** | RNA, 7SL, cytoplasmic 4, pseudogene [Source:HGNC Symbol;Acc:HGNC:10039] |
| ENSG00000264281 | **AC016596.1** |  |
| ENSG00000268350 | **FAM156A** | family with sequence similarity 156 member A [Source:HGNC Symbol;Acc:HGNC:30114] |
| ENSG00000277203 | **F8A1** | coagulation factor VIII associated 1 [Source:HGNC Symbol;Acc:HGNC:3547] |
| ENSG00000006459 | **KDM7A** | lysine demethylase 7A [Source:HGNC Symbol;Acc:HGNC:22224] |

**Supplementary Table S8. Key Resources**

| **REAGENT or RESOURCE** | **SOURCE** | **IDENTIFIER** |
| --- | --- | --- |
| **Antibodies** |  |  |
| p90RSK | Cell signaling technology | Cat #14813 |
| p-p70S6K | Cell signaling technology | Cat # 9204 |
| p70S6K | Cell signaling technology | Cat #9202 |
| MCL1 | Cell signaling technology | Cat # 94296 |
| p-4EBP1 | Cell signaling technology | Cat # 2855 |
| 4EBP1 | Cell signaling technology | Cat # 9644 |
| pS209-eIF4E | Cell signaling technology | Cat # 9741 |
| eIF4E | Cell signaling technology | Cat # sc-271480 |
| BCL2 | Cell signaling technology | Cat # 15071 |
| BCL-XL | Cell signaling technology | Cat # 2764 |
| Caspase 3 | Cell signaling technology | Cat # 9662 |
| GAPDH | Abcam | Cat # ab8245 |
| Actin | Santacruz Biotechnology | Cat # sc-8432 |
| Anti-Mouse IgG, HRP-linked | Santacruz Biotechnology | Cat # sc-516102 |
| Anti-Rabbit IgG, HRP-linked | Santacruz Biotechnology | Cat # sc-2357-CM |
| **Reagents** |  |  |
| m^7^GTP Sepharose Beads | Jena Biosciences | Cat # AC-155S |
| Pierce ECL Western Blotting Substrate | Thermo Fisher Scientific | Cat# 32106 |
| RIPA Lysis and Extraction Buffer | Thermo Fisher Scientific | Cat# 89900 |
| cOmplete™, EDTA-free Protease Inhibitor Cocktail | Sigma Aldrich | Cat # 11873580001 |
| Power SYBR green | Thermo Fisher Scientific | Cat # 4367659 |
| TRI Reagent | Sigma Aldrich | Cat # 93289 |
| Isopropanol | Sigma Aldrich | Cat # 650447 |
| Choloroform | Sigma Aldrich | Cat # 288306-1L |
| PVDF Membrame | Thermo Fisher Scientific | Cat # 88518 |
| NuPAGE™ LDS Sample Buffer (4X) | Thermo Fisher Scientific | Cat # NP0007 |
| 2-Mercaptoethanol | Sigma Aldrich | Cat# M6250 |
| Penicillin/Streptomycin | Thermo Fisher Scientific | Cat# 15140122 |
| DNase I (RNase-free) | New England Biolabs | Cat# M0303S |
| Sucrose | Thermo Fisher Scientific | Cat# AAJ21938A7 |
| High-Capacity cDNA Reverse Transcription Kit | Thermo Fisher Scientific | Cat # 4368814 |
| RPMI 1640 | Thermo Fisher Scientific | 11875119 |
| FBS | Gemini Bio-Products | 100-106H |
| **Biological Samples** |  |  |
| Human primary AML bone marrow mononuclear cells  (AML-29, AML-31) | Dr. Ashkan Emadi | University of Maryland |
| **Cell Lines** |  |  |
| MOLM14 | Dr. Mark Levis | Johns Hopkins University |
| MonoMac6 | Dr. Mark Levis | Johns Hopkins University |
| MV411 | ATCC | Cat# CRL-9591 |
| HL60 | ATCC | Cat# CCL-240 |
| K562 | ATCC | Cat# CCL-243 |
| U937 | ATCC | Cat# CRL-1593.2 |
| **Recombinant DNA** |  |  |
| AML45-luc patient derived cell lines expressing luciferase  plasmid | Dr. Alexander E. Perl  Dr. Martin Carroll | University of Pennsylvania |
| MSCV-derived promoter driving luc2 IRES YFP lentivirus | Dr. Sharyn Baker  St. Jude Viral Vector Core |  |
| pCL20IM-luc-IYFP plasmid | St. Jude Viral Vector Core |  |
| **Software and Algorithms** |  |  |
| GraphPad PRISM 7 | GraphPad Software | https://www.graphpad.com/ |
